# Cargo-eliminated osteosarcoma-derived small extracellular vesicles mediating competitive cellular uptake for inhibiting pulmonary metastasis of osteosarcoma

**DOI:** 10.1186/s12951-024-02636-9

**Published:** 2024-06-22

**Authors:** Shanyi Lin, Longqiang Shu, Yuhang Guo, Ji Yuan, Juntao Zhang, Yang Wang, Yunlong Yang, Ting Yuan

**Affiliations:** 1 Institute of Microsurgery on Extremities, Shanghai Sixth People’s Hospital Affiliated to Shanghai Jiao Tong University School of Medicine, Shanghai, China; 2 Department of Orthopaedic Surgery, Shanghai Sixth People’s Hospital Affiliated to Shanghai Jiao Tong University School of Medicine, Shanghai, China; 3 Peking University People’s Hospital, Beijing, China; 4 Department of Neurosurgery, The First Affiliated Hospital of Wenzhou Medical University, Wenzhou, China

**Keywords:** Tumor derived small extracellular vesicles, Osteosarcoma, Cargo elimination, Lung fibroblast, Pre-metastatic niche

## Abstract

Osteosarcoma (OS) derived small extracellular vesicles (OS-sEVs) have been shown to induce the formation of cancer-associated fibroblasts (CAFs), characterized by elevated pro-inflammatory factor expression and enhanced migratory and contractile abilities. These CAFs play a crucial role in priming lung metastasis by orchestrating the pre-metastatic niche (PMN) in the lung. Disrupting the communication between OS-sEVs and lung fibroblasts (LFs) emerges as a potent strategy to hinder OS pulmonary metastasis. Our previously established saponin-mediated cargo-elimination strategy effectively reduces the cancer-promoting ability of tumor-derived small extracellular vesicles (TsEVs) while preserving their inherent targeting capability. In this study, we observed that cargo-eliminated OS-sEVs (CE-sEVs) display minimal pro-tumoral and LFs activation potential, yet retain their ability to target LFs. The uptake of OS-sEVs by LFs can be concentration-dependently suppressed by CE-sEVs, preventing the conversion of LFs into CAFs and thus inhibiting PMN formation and pulmonary metastasis of OS. In summary, this study proposes a potential strategy to prevent LFs activation, PMN formation in the lung, and OS pulmonary metastasis through competitive inhibition of OS-sEVs’ function by CE-sEVs.

**Supplementary Information:**

The online version contains supplementary material available at 10.1186/s12951-024-02636-9.

## Introduction

Osteosarcoma (OS) is the most common malignant tumor of bone [1, 2]. Since the introduction of systematic chemotherapy, the 5-year survival rate of non-metastatic OS patients has been increased from 20% to over 60%. Pulmonary metastasis is the most troublesome situation in OS patients with an about 16.5% occurrence rate [1, 3]. By contrast, the 5-year survival rate dramatically drops below 20% when pulmonary metastasis occurs [1, 3]. Furthermore, its detection is still a great challenge, which usually results in a delayed treatment. Thus, preventing pulmonary metastasis is a major issue in OS treatment to effectively reduce the mortality. However, up to now, no specific intervention method has been successfully practiced in clinics.

In the past two decades, a pre-metastasis niche (PMN)-based mechanism has been revealed to explain the organotropism of cancer cell migration in metastasis [4]. The PMN is defined as the remolded microenvironment that is favorable for the colonization and outgrowth of tumor cells in specific distant organs [5]. The PMN formation is tightly associated with the interplay between primary tumor cells’ secretomes and the microenvironment in specific organs. This theory has also been proved in pulmonary metastasis development of OS. For example, OS cell secreted glycoprotein ANGPTL2 can promote neutrophil recruitment, thereby perpetuating a chronic inflammation in lung [6]; OS cells-derived COL6A1 remodels the extracellular matrix of local lung microenvironment by promoting inflammatory cytokines and chemokines production [7]; The fusion protein Rab22a-NeoF1 derived from OS cells and its partner PYK2 cause the recruitment of bone marrow-derived macrophages to the lung and M2-type polarization of lung macrophages, and subsequently establish an immunosuppressive microenvironment in lung [8]. Thus, interfering the distant communication between the OS cells and lung microenvironment would have the potential to inhibit the PMN formation and further metastasis.

Small extracellular vesicles (sEVs) are crucial mediators in intercellular communications. These nano-sized (30 –150 nm) vesicles with lipid-bilayer membrane can load various cargos (mainly protein and RNA) of parent cells and effectively transport them to recipient cells and further induce specific responses [9, 10]. Recent studies have identified the vital roles of OS cell-derived sEVs (OS-sEVs) in lung PMN formation. It is found that OS-sEVs exhibits intrinsic lung-tropism [11]. These lung-infiltrating OS-sEVs are mainly internalized by lung fibroblasts (LFs) through an integrin-dependent mechanism [7, 12]. The inner cargos of OS-sEVs convert the LFs into cancer-associated fibroblasts (CAFs) by directly activating crucial signaling in fibroblasts. For example, OS-sEVs carried COL6A1 and TGF-β can activate the NF-κB and TGF/SMAD signaling pathways, respectively [7, 13]. The converted CAFs release cytokines that attract immunosuppressive cells. In addition, their induction of fibronectin deposition and extracellular matrix remodeling can promote tumor cell adhesion and colonization [13–16]. All these changes served as the crucial step during PMN development. Based on these findings, the OS-sEVs mediated tumor-lung communications would be a potential target to inhibit the lung PMN formation.

While, how to specifically inhibit the sEVs mediated communication is still a great challenge. Receptor-mediated endocytosis and membrane fusion are the main uptake mechanisms of sEVs [17, 18]. This implies that the uptake of sEVs may exhibit a saturation kinetic due to the limited distribution of the endocytosis related recognizing and executing proteins such as integrins, clathrins, and cavolins, and the specific sEVs uptake could be competed by certain extraneous nanovesicles. For example, a recent study revealed that the uptake capacity of Kupffer cells towards melanoma cells-derived sEVs (melanoma-sEVs) can be attenuated by pre-administration of extraneous liposomes which has surface phosphatidylserine modification similar to that of melanoma-sEVs [19]. The OS-sEVs show intrinsic lung-tropism and their inner cargos can remold lung microenvironment for PMN development. In our recent study, we had established an inner-cargo eliminating strategy by saponin-assisted cargo leakage of glioma derived sEVs [20]. The intrinsic pro-tumoral ability of these sEVs was dramatically diminished, while their intrinsic tropism for glioma was preserved. After cargo-eliminating, these tumor derived sEVs could be used as highly safe and efficient nanocarriers for glioma chemotherapy [20]. Inspired by these findings, we assume that the cargo-eliminated OS-sEVs (CE-sEVs) that show intrinsic lung-tropism but without PMN inducing ability would have the potential to interfere the in vivo OS-lung communication by competing the cellular uptake of circulating OS-sEVs in lung.

Based on the above statement, herein we have established a competitive cellular uptake strategy mediated by CE-sEVs for preventing pulmonary metastasis. The study revealed that the administration of massive CE-sEVs without PMN inducing ability could compete the cellular uptake (especially LFs) of normal OS-sEVs and further interfere the PMN formation in lung, thus reducing the pulmonary metastasis possibility. For the first time, our study provides an efficient strategy for pulmonary metastasis prevention in OS by targeting sEVs mediated OS-lung communication, and this strategy would have great referential value and application potential in dealing with cancer metastasis.

## Materials and methods

### Cell culture

The human OS cell line MNNG/HOS (MNNG) and the human osteoblast cell line hFOB1.19 were purchased from the American Type Culture Collection (ATCC). The human LFs cell line HFL-1 and human umbilical vein endothelial cell HUVEC were obtained from the Cell Bank of the Chinese Academy of Sciences. MNNG and HUVEC cells were cultured in DMEM medium (Corning, USA); HFL-1 cells were cultured in Ham’s F-12 K medium (Thermo Fisher Scientific, USA); and hFOB1.19 cells were cultured in DMEM/F-12 medium (Thermo Fisher Scientific, USA). All culture media contained 10% fetal bovine serum (FBS) (Thermo Fisher Scientific, USA). The cells were cultured at 37 °C in an atmosphere containing 5% CO₂.

### Isolation of sEVs

Free-sEVs FBS was obtained as reported in a previous study [21]. MNNG, hFOB1.19, and HUVEC cells were cultured in 10 mm cell culture dishes as described above. Once the cells reached 70-80% confluence, the FBS was replaced with Free-sEVs FBS and the cells were further cultured for 48 h. Afterward, the conditioned medium was collected and subjected to sequential centrifugation steps: 300 g for 10 min, 2000 g for 15 min, and 10,000 g for 30 min, to remove dead cells, cell debris, and large extracellular vesicles, respectively. Subsequently, the sEVs from MNNG, hFOB1.19, and HUVEC cells were isolated by double ultracentrifugation at 100,000 g for 70 min at 4 °C and stored at -80 °C.

### sEVs cargos elimination

To eliminate the cargos of OS-sEVs, saponin treatment was applied as per our previous study [20]. Briefly, 1 × 10^10^ particles of OS-sEVs were treated with 1 mL 0.2% w/v of saponin for 30 min at room temperature (RT), followed by addition of 30 mL sterile PBS and ultracentrifugation twice at 100,000 g for a total duration of 140 min at a temperature of 4℃ to precipitate CE-sEVs.

### Transmission electron microscopy (TEM)

A total of 10 µL OS-sEVs or CE-sEVs solution which contain 2 × 10^8^ particles were put on a formvar carbon-coated grid (300 meshes), then, leave at RT for 20 min. After that, wash the grid and fixed by 1% glutaraldehyde for 5 min, followed by washing it in water and staining it in 2% saturated aqueous uranyl oxalate for 5 min. Finally, after drying for 10 min, the grid was imaged in TEM (Hitachi, Japan).

### Particle concentration and size distribution

The size distribution and concentration of OS-sEVs or CE-sEVs were detected by a nanoflow cytometer (nanoFCM, China). For particle concentration detection, firstly, a standard nanoparticle with a diameter of 200 nm and a concentration of 1.58 × 10^8^/mL was used for quantification. Next, the sEVs samples were measured by nanoflow. Finally, particle concentration of samples was calculated via the recorded particle number of the samples and the standard nanoparticles. For size distribution detection, firstly, a set of standard nanoparticles with a diameter of 68, 91, 113, and 155 nm were used to create the standard curve. Next, the sEVs samples were measured by nanoflow. Finally, the size distribution of samples was fitted to the standard curve and obtained.

### Detection of total sEVs protein

For BCA assay, the OS-sEVs (1 × 10^10^ particles) and CE-sEVs (1 × 10^10^ particles) were lysed using RIPA buffer (EpiZyme, China) and followed by detection using the Pierce BCA Protein Assay Kit (Beyotime Biotechnology, China) according to the manufacturer’s instructions. The samples’ absorbance was measured using a Bio-Rad plate reader at a wavelength of 562 nm, and the protein concentration of each sample was calculated using a standard curve. For sliver staining, the total proteins from OS-sEVs (1 × 10^10^ particles) and CE-sEVs (1 × 10^10^ particles) were extracted using RIPA solution (EpiZyme, China) following standard protocols and separated by electrophoresis on SDS-PAGE. Following electrophoresis, the gel was immersed in a fixative solution containing 50 mL ethanol, 10 mL acetic acid, and 40 mL Milli-Q grade pure water and incubated for 60 min at room temperature with shaking at a speed of 60–70 rpm. Subsequently, silver staining was performed according to the manufacturer’s instructions (Beyotime Biotechnology, China).

### Detection of total sEVs RNA

For RNA fluorescence staining, The OS-sEVs (1 × 10^10^ particles) and CE-sEVs (1 × 10^10^ particles) were suspended in PBS for detection of total RNA in sEVs using the SYTO® RNA Select™ Green Fluorescent Cell Stain kit (Thermo Fisher Scientific, USA) as per instructions. The samples’ absorbance was measured using a Bio-Rad plate reader at a wavelength of 530 nm. For RNA enrichment analysis, the total RNA from OS-sEVs (1 × 10^10^ particles) and CE-sEVs (1 × 10^10^ particles) was quantified using Agilent Bioanalyzer 2100 (Agilent Technologies, Santa Clara, CA, USA) and expressed as fluorescence units (FU) / nucleotide (nt).

### Western blot analysis and reagents

Total proteins of sEVs and cells were extracted by RIPA solution (EpiZyme, China) according to standard procedures. For western blot, firstly, the collected total proteins were separated by electrophoresis in SDS-PAGE. Next, proteins were transferred to a PVDF membrane and the nonspecific binding sites were blocked by 5% milk at RT for 2 h. Target proteins were probed via incubation in the primary antibody solution at 4 °C overnight. The primary antibodies used: CD9 (1:1500, Abcam, ab92726), CD63 (1:1000, Abcam, ab134045), TSG101 (1:1000, Santa Cruz Biotechnology, sc7964), GM130 (1:1000, Abcam, ab52649), TGF-β (1:1000, Abcam, ab215715).

### RNA extraction and real-time quantitative PCR (RT-qPCR)

Total RNA from cell lines was extracted by TRIzol reagent (Thermo Fisher Scientific, USA) and then reverse transcribed to cDNA according to standard procedures (Thermo Fisher Scientific, USA). The relative gene expression levels were measured on an ABI Prism 7900HT real-time system (Applied Biosystems) and calculated by the 2^−ΔΔCt^ approach. All primers are shown in Supplementary Table 1.

### Cell counting Kit-8 (CCK-8) assay

MNNG cells was seed on a 96-well plate at a density of 5,000 cells/well (*n* = 3). Then, sEVs were treated. Briefly, 1 × 10^9^ particles/mL OS-sEVs or CE-sEVs were incubated with MNNG cells. The cell viability was measured by a CCK-8 kit (Dojindo) at 0 h, 24 h, 48 h, and 72 h according to the manufacturer’s protocol. The absorbance of each cell sample was detected at 450 nm by a plate reader (Bio-Rad, USA).

### Transwell assay

The function of OS-sEVs and CE-sEVs on the migration of cancer cells was evaluated by transwell assay. Total 5 × 10^4^ MNNG cells together with 1 × 10^9^ particles OS-sEVs or CE-sEVs were seeded in 0.2 mL serum-free medium in the upper chamber, 700 µL medium containing 10% FBS was added into lower chamber. After 24 h, the cells migrated to the bottom of the chamber were fixed and stained with 1% crystal violet solution before being observed with a microscope.

### Wound healing assay

HFL-1 cells were cultured in a 6-well plate (*n* = 3), when confluent up to 100%, the cells were scraped in a straight line to create a “scratch” with a 200 µL pipette tip, take the images as the 0 h. Then, treated the cells with 1 × 10^9^ particles/mL OS-sEVs, 1 × 10^9^ particles/mL CE-sEVs or 1 × 10^10^ particles/mL CE-sEVs, after that, the images were taken at 12 h and 24 h. The wound closure percentage was calculated as the formula:

$$\frac{\text{t}\text{h}\text{e} \,\text{m}\text{i}\text{g}\text{r}\text{a}\text{t}\text{e}\text{d}\, \text{c}\text{e}\text{l}\text{l}\, \text{s}\text{u}\text{r}\text{f}\text{a}\text{c}\text{e}\, \text{a}\text{r}\text{e}\text{a}}{\text{t}\text{o}\text{t}\text{a}\text{l}\, \text{s}\text{u}\text{r}\text{f}\text{a}\text{c}\text{e} \,\text{a}\text{r}\text{e}\text{a}}\times 100\text{\%}.$$

### Collagen contraction assays

Firstly, 200 µL Type 1 Rat Tail Collagen (Solarbio) was mixed with 12 µL 0.1 mol/L NaOH and 23 µL 10×PBS. Subsequently, the mixture was combined with a cell suspension containing 4 × 10^5^ HFL-1 cells (760 µL), and added to individual wells in a 24-well plate (500 µL/well). Finally, after polymerizing at 37 °C for 30 min, add another 500 µL medium containing OS-sEVs (1 × 10^9^ particles), CE-sEVs (1 × 10^9^ particles) or CE-sEVs (1 × 10^10^ particles). The images were taken at 0 h, 24 h, and 72 h. The contraction was calculated as the formula:

$$\frac{\text{w}\text{e}\text{l}\text{l}\, \text{s}\text{u}\text{r}\text{f}\text{a}\text{c}\text{e}\, \text{a}\text{r}\text{e}\text{a}-\text{g}\text{e}\text{l}\, \text{s}\text{u}\text{r}\text{f}\text{a}\text{c}\text{e}\, \text{a}\text{r}\text{e}\text{a}}{\text{w}\text{e}\text{l}\text{l}\, \text{s}\text{u}\text{r}\text{f}\text{a}\text{c}\text{e}\, \text{a}\text{r}\text{e}\text{a}}\times 100\text{\%}.$$

### RNA-seq and analysis

TRIzol (Thermo Fisher Scientific, USA) was used to extract total RNA from the HFL-1 cells treated with PBS, OS-sEVs (1 × 10^9^ particles/mL), or CE-sEVs (1 × 10^9^ particles/mL). Subsequently, a TruSeq™ RNA Sample Preparation Kit (Illumina, USA) was utilized to construct paired-end libraries in accordance with standard guidelines. The mRNA was fragmented and reverse transcribed into first strand cDNA. Then, the second strand cDNA was synthesized using DNA polymerase I and RNase H. The resulting cDNAs were subjected to A-tailing and adapter ligation, followed by purification and PCR enrichment to generate the final cDNA library. Library construction and sequencing were performed by Sinotech Genomics Co., Ltd. (Shanghai, PRC). Differentially expressed genes were identified based on a false discovery rate of less than 5% and fold changes greater than 1.5 or less than 0.67. All cell lines underwent three replicates to ensure accuracy.

### In vitro sEVs internalization assays

The staining of sEVs was performed as follows: sEVs at a concentration of 1 × 10^10^ particles/mL were labeled with 10 µM DiR or DiO (Thermo Fisher Scientific, USA) at 37℃ for 30 min. Subsequently, the labeled sEVs were filtered through a 0.22 μm membrane and washed twice with PBS. Finally, centrifugation at high speed was performed to separate the stained sEVs from the supernatant.

For in vitro sEVs internalization assays, the DiO-labeled OS-sEVs or DiO-labeled CE-sEVs were added into culture medium and incubated with HFL-1 cells for 12 h. Next, the cells were fixed with 4% paraformaldehyde for 15 min, permeabilized for 10 min, and the nuclei were stained with 4′, 6-diamidino-2- phenylindole (DAPI, Beyotime Biotechnology, China) prior to the image capture using the fluorescence microscope (Leica, Germany). For flow cytometry analysis, at the end of culture, the cells were washed twice with PBS and then suspended in PBS before being subjected to detection using a flow cytometer (Cytoflex, USA).

For in vitro competitive cellular uptake assay mediated by CE-sEVs, HFL-1 cells were treated with a combination of DiR-labeled OS-sEVs and non-labeled CE-sEVs. The CE-sEVs were added to the culture medium at final concentrations of 0 particles/mL, 5 × 10^9^ particles/mL, and 1 × 10^10^ particles/mL in different groups, while the OS-sEVs were present at a final concentration of 1 × 10^9^ particles/mL in all groups. In contrast, for in vitro competitive cellular uptake assay mediated by sEVs derived from different cell types rather than OS, we employed a similar approach. The Proteinase K-treated OS-sEVs (P-OS-sEVs) were obtained as described by Gustafson et al., and the phosphatidylserine-based liposomes were prepared according to the instructions of the liposome kit (Sigma-Aldrich, USA). Then, HFL-1 cells were treated with a combination of DiO-labeled OS-sEVs and non-labeled hFOB-sEVs, HUVEC-sEVs, P-OS-sEVs, and liposomes. The final concentration of OS-sEVs was 1 × 10^9^ particles/mL, while the hFOB-sEVs, HUVEC-sEVs, P-OS-sEVs, and liposomes were 1 × 10^10^ particles/mL. Finally, after culturing for 12 h, the cells were fixed with 4% paraformaldehyde for 15 min, permeabilized for 10 min, stained with DAPI, and imaged using a confocal fluorescence microscope (Leica, Germany). For flow cytometry analysis, at the end of the culture period, the cells were washed twice with PBS and suspended in PBS before being analyzed using a flow cytometer (Cytoflex, USA).

### Stable cell line construction

The lentivirus shuttle plasmids containing full-length luciferase were co-transfected into HEK293T cells with lentivirus packing vectors. After 48 h, the supernatant of HEK293T, which contains lentivirus was collected, purified, and performed titer determination. Then, MNNG cells were infected by the collected lentivirus at the MOI = 10. The positive cells were (MNNG-luc) obtained by 1.0 µg/mL puromycin selection after 72 h after infection.

### Animal experiments

Female nude mice aged 4–6 weeks were procured from the Laboratory Animal Research Center of Shanghai Sixth People’s Hospital, with all procedures being sanctioned by the Animal Research Committee of Shanghai Sixth People’s Hospital.

To investigate the impact of sEVs on OS cells, we utilized a subcutaneous tumor model. Briefly, following anesthesia with pentobarbital sodium, 200 µL of cell suspension containing 1 × 10^6^ MNNG cells were inoculated into the flank of nude mice. The mice were then randomly allocated into three groups and administered with PBS (100 µL), OS-sEVs (1 × 10^10^ particles/mL, 100 µL), and CE-sEVs (1 × 10^10^ particles/mL, 100 µL) three times a week, respectively. These mice were sacrificed on day 18, and the tumors were collected. The volume of tumors was calculated using the formula length (mm) × width (mm)^2^/2.

To confirm the in vivo co-localization of sEVs and LFs, OS-sEVs or CE-sEVs were labeled with DiR. Then, 100 µL sEVs suspension (1 × 10^10^ particles/mL) were intravenously injected into mice. After 24 h, the mice were sacrificed, the lungs were harvested and fixed in PFA at 4 °C for 12 h, the dehydration with 20%, 30%, and 35% sucrose solutions at 4 °C, respectively. After embedding with OCT, lung was cut into sections and follow by incubation with primary antibodies against S100A4. The images were taken by confocal fluorescence microscope (Leica, Germany).

To investigate the competitive cellular uptake capability mediated by CE-sEVs, 100 µL suspension of DiR-labeled OS-sEVs (1 × 10^10^ particles/mL) was intravenously administered into mice. The mice were then randomly allocated into three groups and administered with blank, PBS (100 µL), and CE-sEVs (1 × 10^11^ particles/mL, 100 µL), respectively. After 24 h, the mice were euthanized, their main organs harvested, and the accumulation of DiR-labeled OS-sEVs in lung was evaluated by ex vivo bioluminescent imaging (BLI) (Caliper, USA).

For the PMN formation study, the nude mice were firstly randomly assigned into three experimental groups, the control group was injections of OS-sEVs (1 × 10^10^ particles/mL, 100 µL) every other day, the PBS group with OS-sEVs (1 × 10^10^ particles/mL, 100 µL) and PBS (100 µL) every other day, and the CE-sEVs group was injections of OS-sEVs (1 × 10^10^ particles/mL, 100 µL) and CE-sEVs (1 × 10^11^ particles/mL, 100 µL) every other day. On day 7, mice were sacrificed and the fibroblast activation and PMN formation markers on the lung was evaluated using immunofluorescence (IF). For spontaneous metastasis assay, 1 × 10^6^ MNNG cells were suspended in 20 µL of PBS and injected into the medullary cavity of the tibia. The mice were then divided into three groups and treated with blank, PBS (100 µL), and CE-sEVs (1 × 10^11^ particles/mL, 100 µL) three times per week. On day 14, mice were sacrificed and fibroblast activation and PMN formation markers on the lung was evaluated using IF.

Two metastasis models were employed to assess the effect of CE-sEVs in OS metastasis. In the experimental metastasis model, three groups were established. The control group was non-pretreated, PBS group was pretreated with OS-sEVs (1 × 10^10^ particles/mL, 100 µL) + PBS (100 µL), and CE-sEVs group was pretreated with OS-sEVs (1 × 10^10^ particles/mL, 100 µL) + CE-sEVs (1 × 10^11^ particles/mL, 100 µL), and each group was administered once every three days. The MMNG cells (1 × 10^6^ cells) were administered via the tail vein on day 12, and the mice were subsequently euthanized on day 28. Their lungs were then excised for observation of lung metastasis. For spontaneous metastasis model, an orthotopic OS model was established by injecting 1 × 10^6^ MNNG cells (20 µL) into the tibia. The mice were then randomly allocated into three groups for three times a week intervention, i.e., control group (blank), PBS group (PBS 100 µL), and CE-sEVs group (1 × 10^11^ particles/mL, 100 µL). At the end of the fourth week, the mice were sacrificed and their lungs were collected to observe metastatic activity.

### Statistical analyses

The data were analyzed by SPSS 25.0 software and are presented as the mean ± SD. The differences between the experimental and control groups were analyzed by two-tailed Student’s t test. ns indicates *P* > 0.05, * indicates *P* < 0.05, ** indicates *P* < 0.01, and *** indicates *P* < 0.001, # indicates *P* < 0.0001.

## Results

### Preparation and characterization of CE-sEVs

**Fig. 1:**
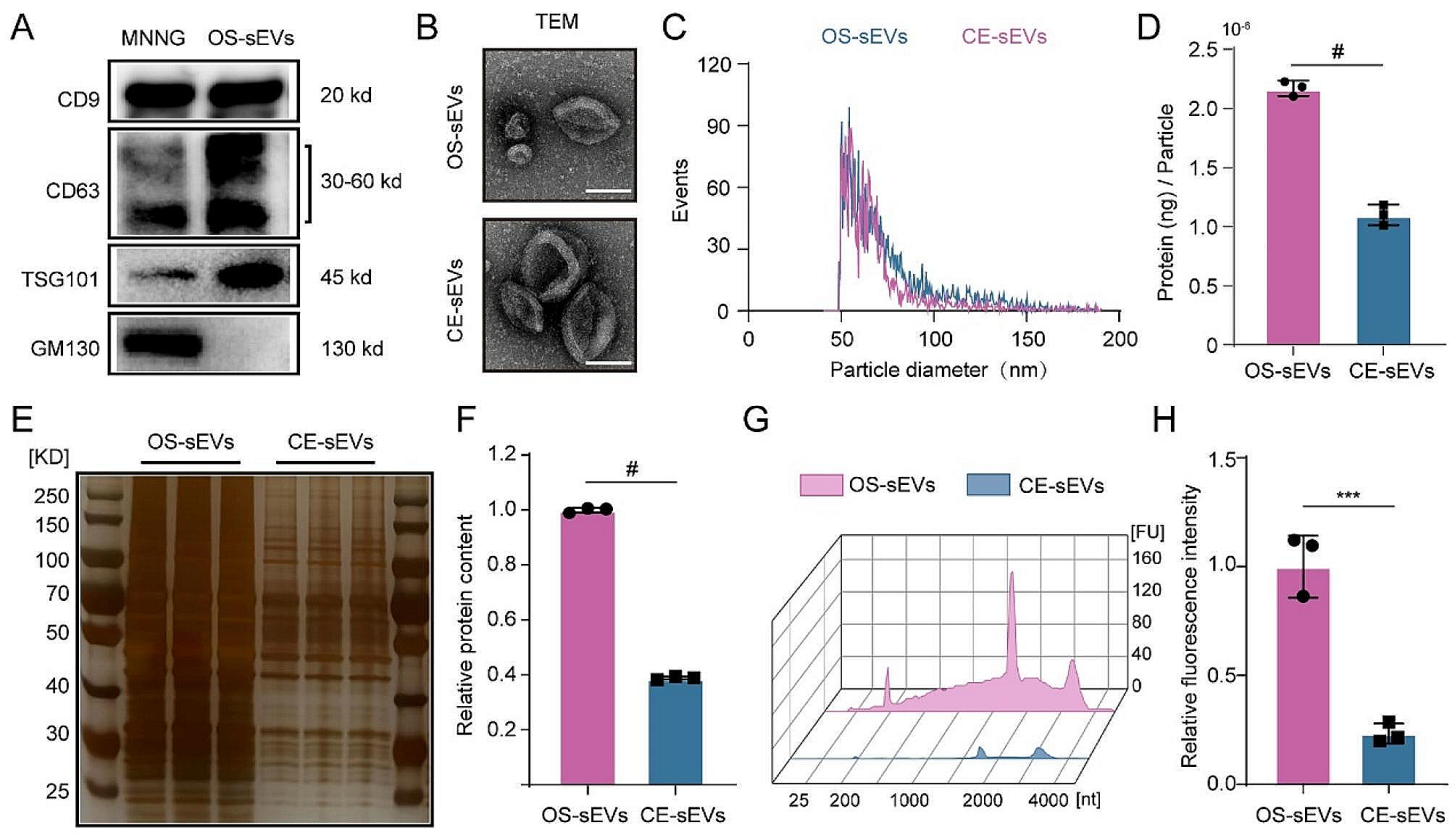
Characterization of OS-sEVs and CE-sEVs. **(A)** Western blot analysis of sEVs characteristic markers (CD9, CD63, and TSG101) and non-sEVs marker GM130 in MNNG cells and OS-sEV. **(B)** Representative TEM images of OS-sEVs and CE-sEVs. Scale bar: 100 nm. **(C)** Particle size distribution of OS-sEVs and CE-sEVs. **(D)** Quantification of the mean protein concentration per particle of OS-sEVs and CE-sEVs (*n* = 3). **(E)** Sliver staining image of total proteins in OS-sEVs (1 × 10^10^ particles) and CE-sEVs (1 × 10^10^ particles) and **(F)** the quantification of the relative protein content of OS-sEVs and CE-sEVs (*n* = 3). **(G)** RNA enrichment analysis depicted in FU per nt of total RNA contents in OS-sEVs (1 × 10^10^ particles) and CE-sEVs (1 × 10^10^ particles). **(H)** Total RNA contents in OS-sEVs (1 × 10^10^ particles) and CE-sEVs (1 × 10^10^ particles) by SYTO™ RNA staining (*n* = 3). *** *P* < 0.001; # *P* < 0.0001

The OS-sEVs were isolated from the OS cell culture medium using standard differential centrifugation methods. As shown in Fig. 1A, the isolated OS-sEVs displayed characteristic markers of CD9, CD63, and TSG101, while lacking expression of GM130, which was positively expressed in the OS cells. Additionally, these sEVs exhibited a cup-shaped structure (Fig. 1B), and their size ranged from 50 nm to 150 nm (Fig. 1C), confirming their identity as sEVs. Subsequently, the saponin-mediated cargo elimination was applied to these OS-sEVs to yield CE-sEVs, following our previously established protocol [20]. TEM imaging and nanoflow cytometry analysis revealed that CE-sEVs exhibited morphological features (cup-shaped) and a size distribution (50 –150 nm) comparable to OS-sEVs (Fig. 1B and C). Taken together, these characteristic results suggest that the main physical and membrane properties of CE-sEVs are similar to those of OS-sEVs.

Proteins and RNAs are the predominant functional cargos in sEVs. Therefore, we conducted comprehensive assessments to identify the saponin mediated cargo-elimination of OS-sEVs. The BCA results showed that saponin treatment results in a 49.3% ± 4.0% eliminating efficacy in protein content of CE-sEVs (1.10 × 10^− 6^ ± 0.08 × 10^− 6^ ng/particle) compared to the OS-sEVs (2.17 × 10^− 6^ ± 0.06 × 10^− 6^ ng/particle) (Fig. 1D). Moreover, silver staining was employed to visualize the total proteins in both the OS-sEVs and CE-sEVs. The result (Fig. 1E and F) clearly demonstrated a noticeable reduction in the protein band of CE-sEVs groups (61.1% ± 0.5% eliminating efficacy). Next, the eliminating efficacy of RNA were further evaluated. We initially conducted RNA enrichment analysis by quantifying RNA content as fluorescence units (FU). This analysis revealed a significant decrease in RNA levels ranging from 25 nt to 4000 nt in the CE-sEVs (Fig. 1G). Subsequently, the total RNA was stained in both the OS-sEVs and CE-sEVs using SYTO™ RNA Select Green dye. The results confirmed that saponin treatment leads to the elimination of 76.54% ± 4.63% of RNA in the OS-sEVs (Fig. 1H). Collectively, these data demonstrated high effectiveness in the removal of OS-sEVs’ original cargos by saponin treatment. In addition, the main physiochemical properties of OS-sEVs were preserved during saponin-mediated cargo elimination.

### Diminished effects of CE-sEVs on primary tumor progression

**Fig. 2:**
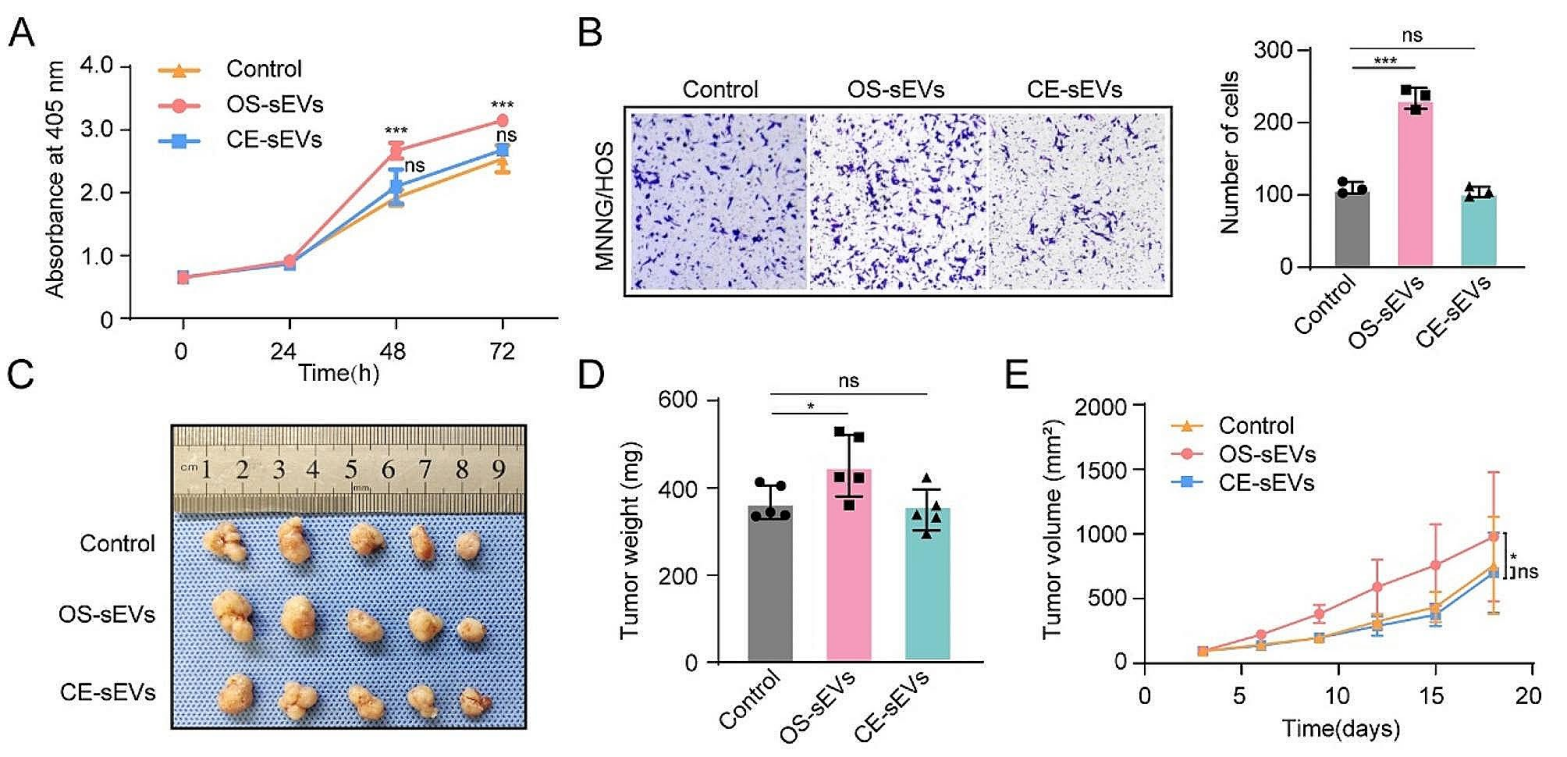
CE-sEVs exhibit no tumor-promoting ability. **(A)** CCK-8 assay detects the proliferation of MNNG cells after treated with OS-sEVs and CE-sEVs, the results confirm that OS-sEVs promoted OS cell proliferation, while CE-sEVs did not have such effect (*n* = 3). **(B)** Transwell assay detects the migration of MNNG cells after treated with OS-sEVs and CE-sEVs, the results confirm that OS-sEVs promoted OS cell migration, while CE-sEVs did not have such effect (*n* = 3). **(C)** Tumor resection images after intravenous intervention with OS-sEVs (*n* = 5), CE-sEVs (*n* = 5), and control (*n* = 5) in the nude mouse subcutaneous tumor model, administered three times per week. **(D)** Quantitation of tumor weight (mean ± SD). **(E)** Quantitation of tumor volume (mean ± SD). ns *P* > 0.05; * *P* < 0.05; *** *P* < 0.001

Tumor derived sEVs are usually reported to promote primary tumor progress, leading to the potential biosafety concern when using these vesicles. Therefore, to ensure the administrating safety of CE-sEVs, we conducted a series of careful assessments to evaluated their pro-tumoral functions and compared them with OS-sEVs. The results of the CCK-8 assay revealed that treatment with OS-sEVs significantly enhanced the proliferation of OS cells. As depicted in Fig. 2A, the OS cell growth rate in the OS-sEVs group was determined to be 1.39 ± 0.07-fold and 1.24 ± 0.02-fold higher than that of the control group at 48 and 72 h, respectively. Conversely, it was observed that CE-sEVs did not elicit similar effects, with cell growth rates of 1.09 ± 0.14-fold and 1.06 ± 0.03-fold compared to the control group at 48 and 72 h, respectively. Migration ability also showed a similar trend. Compared to the control group, the OS cells co-incubated with OS-sEVs showed a 2.13 ± 0.13-fold increase in the number of migratory cells. However, CE-sEVs did not demonstrate the ability to promote OS cell migration as the ratio of migrated cells in the CE-sEVs group vs. control group was 0.95 ± 0.06) (Fig. 2B). Moreover, to evaluate the in vivo biosafety of CE-sEVs, subcutaneous xenograft tumor models were established. Commencing from one week post-tumor inoculation, the OS-sEVs group demonstrated a significantly elevated tumor growth rate in comparison to the other two groups (Fig. 2E). At the end of the experiment, it was observed that the OS-sEV group had the highest average tumor weight: 451.30 ± 70.84 mg. In contrast, no significant difference in tumor average weight was observed between the CE-sEV group (356.21 ± 47.18 mg) and the control group (367.09 ± 38.51 mg). (Figure 2C and D). The results demonstrated that frequently administrated CE-sEVs did not promote tumor growth in vivo. In summary, these findings indicated an acceptable in vivo safety of frequent CE-sEVs administration under OS situation.

#### CE-sEVs mediated competitive cellular uptake

**Fig. 3:**
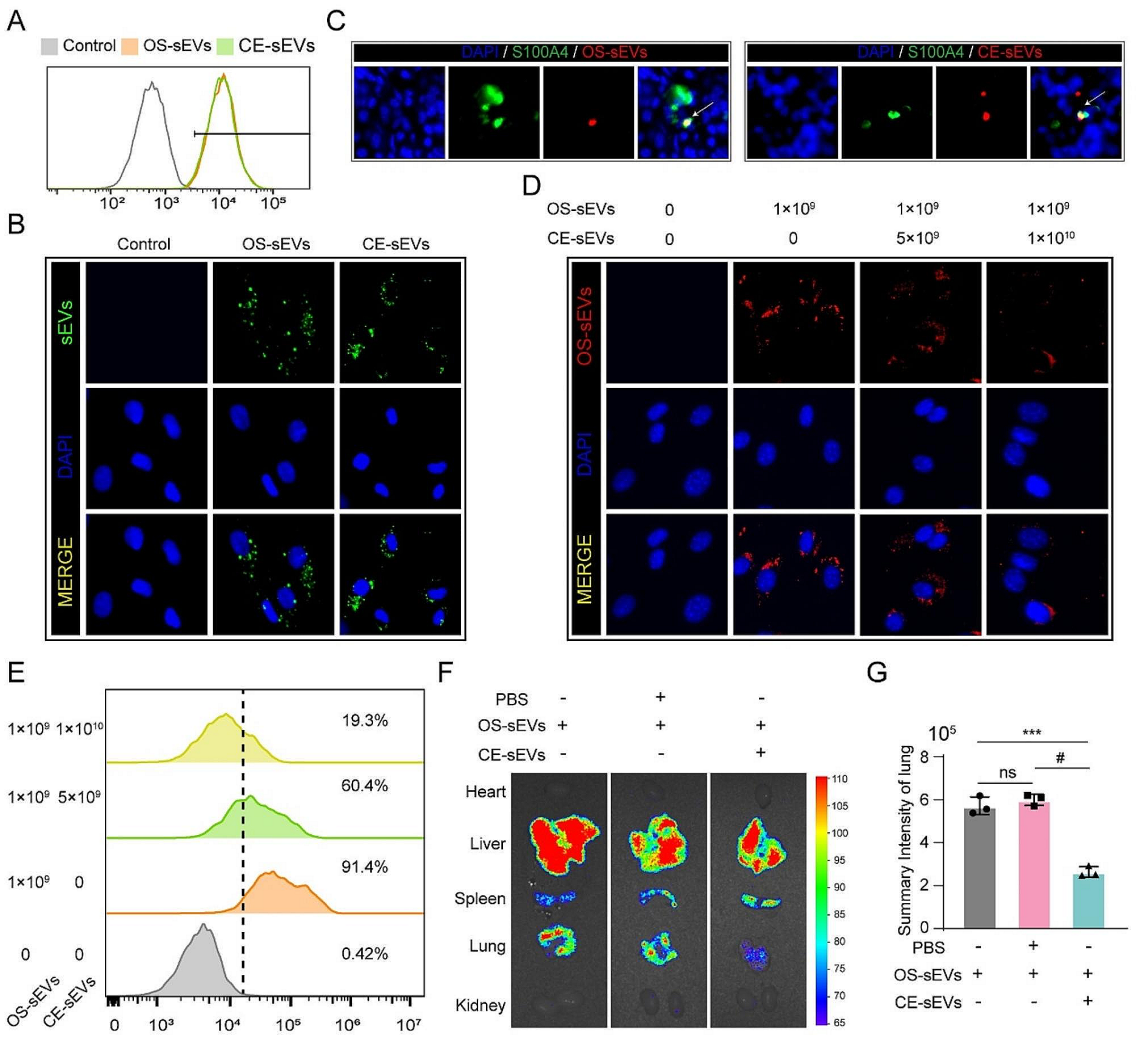
CE-sEVs competitively suppressed the uptake of OS-sEVs by LFs. **(A)** Representative images of HFL-1 cells treated with DiO-labelled OS-sEVs or DiO-labelled CE-sEVs detected by flow cytometry. **(B)** Representative images of the HFL-1 cells uptake of DiO-labelled OS-sEVs (green) or DiO-labelled CE-sEVs (green), CE-sEVs show similar internalized ability to OS-sEVs. **(C)** IF images of OS-sEVs (red, DiR) and CE-sEVs (red, DiR) co-localization with LFs (green, S100A4). **(D)** IF show the uptake efficiency of HFL-1 towards OS-sEVs in different CE-sEVs concentration (0 particles/mL, 5 × 10^9^ particles/mL, and 1 × 10^10^ particles/mL in group 2, group 3, and group 4, respectively). **(E)** Flow cytometry show the uptake efficiency of HFL-1 towards OS-sEVs in different CE-sEVs concentration (0 particles/mL, 5 × 10^9^ particles/mL, and 1 × 10^10^ particles/mL in group 2, group 3, and group 4, respectively). **(F)** Representative ex vivo fluorescence images of main organs. Mice were intravenously injected with DiR-labeled OS-sEVs, followed by interventions with blank, PBS, and CE-sEVs, respectively. After 24 h, the main organs were harvested for ex vivo fluorescence observation. **(G)** Statistical analysis of the FI in lung (*n* = 3). ns *P* > 0.05; *** *P* < 0.001; # *P* < 0.0001

In this investigation, we propose a competitive cellular uptake strategy aimed at disrupting the sEVs-based communication between OS cells and the lung microenvironment. Current researches identified LFs as the primary targets for OS-sEVs. To ensure a highly competitive uptake, CE-sEVs should at least possess a comparable targeting and internalization ability as OS-sEVs. To elucidate this, HFL-1 cells were incubated with DiO-labeled OS-sEVs (1 × 10^9^ particles/mL) or DiO-labeled CE-sEVs (1 × 10^9^ particles/mL). The cytometry analysis results demonstrated similar positive rates in HFL-1 cells treated with OS-sEVs and CE-sEVs after 12-hour incubation (Fig. 3A). Additionally, fluorescent images of HFL-1 cell uptake also revealed a comparable fluorescence intensity (FI) in cells treated with CE-sEVs and OS-sEVs (Fig. 3B). These findings support that the CE-sEVs exhibit similar LFs internalization ability, and saponin treatment did not compromise the targeting and internalization ability of OS-sEVs. Next, we further confirmed the lung targeting ability of CE-sEVs in vivo. Based on the results presented in Fig. S1A and S1B, mice injected with 1 × 10^9^ particles sEVs exhibited a more apparent fluorescence increase over time and reached a significant level allowing visible observation at 24 h. Thus, these parameters (24 h after injection and 1 × 10^9^ particles) were chosen for in vivo observation. Subsequently, 1 × 10^9^ particles of DiR-labeled OS-sEVs or DiR-labeled CE-sEVs were intravenously injected into the mice and the lung tissues were collected 24 h later. The immunofluorescent staining results clearly demonstrated that CE-sEVs retained the same targeting ability for LFs in vivo as OS-sEVs, as illustrated in Fig. 3C, with both CE-sEVs and OS-sEVs co-localizing with S100A4-positive LFs.

Subsequently, the competitive cellular uptake was conducted, the HFL-1 uptake of DiR-labelled OS-sEVs under co-incubation with varied concentration of CE-sEVs was evaluated. Flow cytometry analysis results demonstrated that HFL-1 cells had an over 90% OS-sEVs uptake efficacy in the absence of CE-sEVs. However, when CE-sEVs were present at concentrations of 5 × 10^9^ particles/mL (5-fold to OS-sEVs) and 1 × 10^10^ particles/mL (10-fold to OS-sEVs), the internalization efficiency of OS-sEVs dramatically decreased to approximately 60% and 20%, respectively (Fig. 3E). Furthermore, additional fluorescence imaging results visually revealed a reduction in the number of internalized OS-sEVs with increasing concentrations of CE-sEVs (Fig. 3D). These results clearly demonstrated the massive co-existed CE-sEVs could effectively compete and thus inhibit the uptake of OS-sEVs by LFs.

To further validate our hypothesis, we investigated whether sEVs derived from different cell types, membrane protein degraded OS-sEVs, or artificial liposomes could suppress the uptake of OS-sEVs by lung LFs. Specifically, we assessed the uptake of DiO-labeled OS-sEVs by HFL-1 cells, when co-incubated with four distinct types of sEVs: sEVs derived from hFOB1.19 (hFOB-sEVs), sEVs derived from HUVEC (HUVEC-sEVs), Proteinase K-treated OS-sEVs (P-OS-sEVs), and a commercial liposome. As depicted in Fig. S2, after 12 h of incubation, the fluorescent images of HFL-1 cells exhibited comparable FI among different groups (Fig. S2A). Similarly, the flow cytometry analysis revealed similar positive rates in HFL-1 cells across different groups (Fig. S2B). These results indicate that the co-existence of hFOB-sEVs, HUVEC-sEVs, P-OS-sEVs, and liposomes with OS-sEVs does not hinder or inhibit the uptake of OS-sEVs by HFL-1 cells, potentially lacking the capacity to prevent the pulmonary metastasis of OS cells.

We further hypothesized that CE-sEVs could inhibit the lung accumulation of OS-sEVs in vivo. To assess this hypothesis, nude mice were intravenously injected with 1 × 10^9^ particles of DiR-labeled OS-sEVs, followed by intravenous administration of either PBS (100 µL) or CE-sEVs (1 × 10^10^ particles). After 24 h, the major organs were extracted for fluorescent imaging, and the results are presented in Fig. 3F. In all samples injected with DiR-labeled OS-sEVs, the liver and spleen exhibited a strong fluorescent signal, likely attributable to the uptake of sEVs by the reticuloendothelial system (Fig. 3F, Fig. S1C). Notably, the lung displayed the most intense FI apart from the liver and spleen. Quantitative analysis of lung FI confirmed that PBS treatment did not alter the accumulation of OS-sEVs in the lung, as indicated by the ratio of lung FI in the PBS group compared to the control group (1.05 ± 0.05). However, when comparing the lung FI of the CE-sEVs group with that of the control group, it was observed that the presence of 10-fold CE-sEVs resulted in a 53.8% ± 4.3% reduction in the aggregation of OS-sEVs in the lung (Fig. 3G). These results collectively demonstrate that CE-sEVs can effectively interfere with the lung accumulation of OS-sEVs.

### CE-sEVs induced suppression of LFs activation

**Fig. 4:**
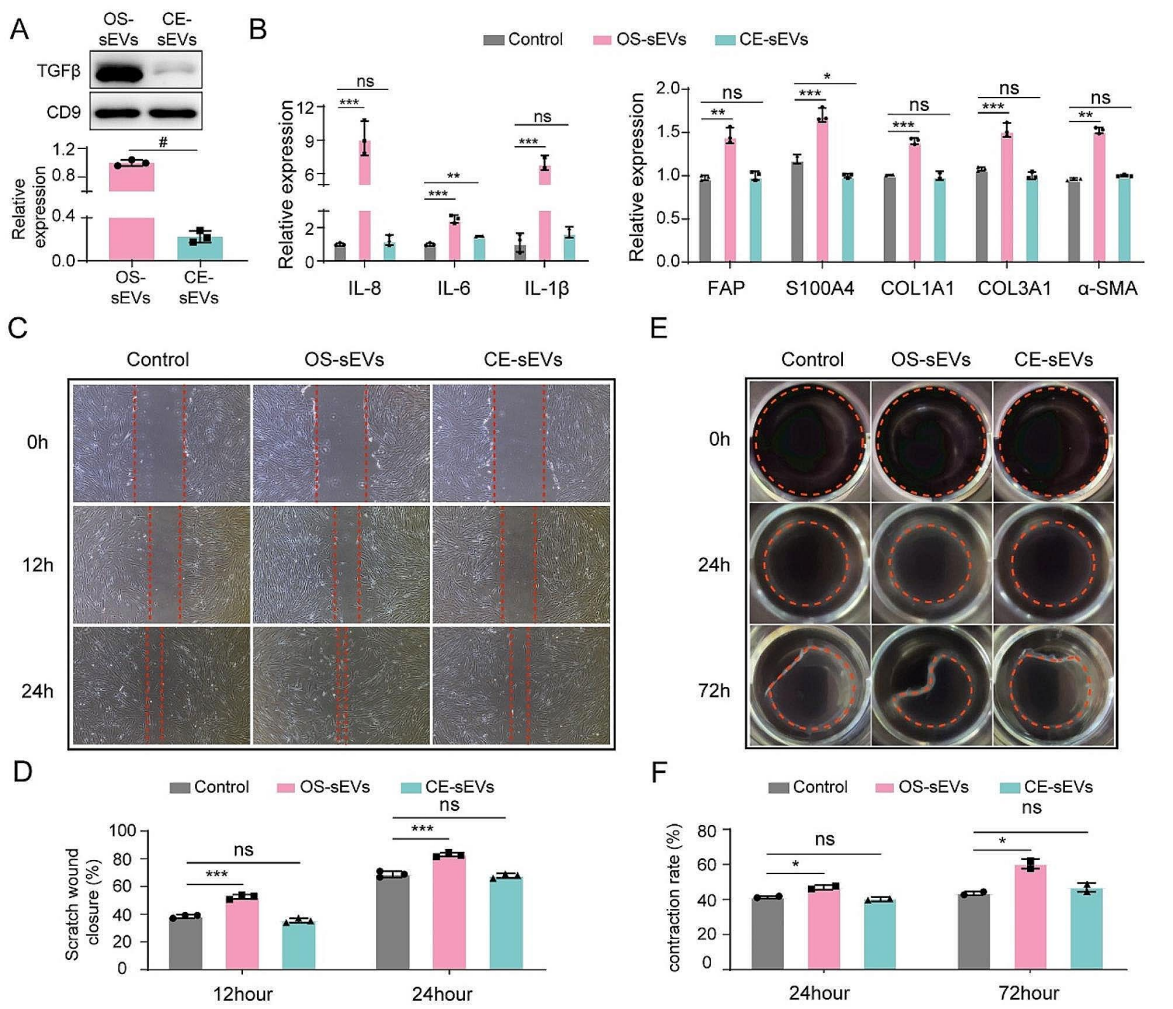
CE-sEVs exhibit no LFs-activating ability. **(A)** Western blot analysis the expression of TGF-β, a key mediator of LFs activation, in OS-sEVs and CE-sEVs (*n* = 3). **(B)** RT-qPCR analysis the expression of the genes associated with LFs activation in HFL-1 cells treated with OS-sEVs or CE-sEVs for 24 h. **(C)** Representative images of wound healing analysis of HFL-1 cells cultured with OS-sEVs or CE-sEVs for 0 h, 12 h, and 24 h. **(D)** quantification of the migration rate of HFL-1 cells cultured with OS-sEVs or CE-sEVs for 0 h, 12 h, and 24 h (*n* = 3). **(E)** Representative images of collagen matrix contraction analysis of HFL-1 cells cultured with OS-sEVs or CE-sEVs for 0 h, 24 h, and 72 h and **(F)** quantification of the contraction rate of HFL-1 cells cultured with OS-sEVs or CE-sEVs for 0 h, 24 h, and 72 h (*n* = 2). ns *P* > 0.05; * *P* < 0.05; ** *P* < 0.01; *** *P* < 0.001; # *P* < 0.0001

OS-sEVs’ cargos have the function to activate LFs to facilitate the formation of PMN. Here, we hypothesized that this effect could be diminished through cargo-elimination. Next, we investigated the effect of CE-sEVs in activating LFs. According to the related researches, the OS-sEVs carried TGF-β is a major factor for LFs activation [13]. Thus, the expression eliminated efficacy of TGF-β was evaluated. As shown in Fig. 4A, the content of TGF-β in CE-sEVs was reduced by 83.1% ± 4.1% after saponin treatment compared to OS-sEVs. This reduction indicates the effective removal of the key components in OE-sEVs that activate LFs. Subsequently, HFL-1 cells were exposed to OS-sEVs (1 × 10^9^ particles/mL) or CE-sEVs (1 × 10^9^ particles/mL) to assess the potential of CE-sEVs in activating of LFs. The upregulated expression of LFs activation markers, including FAP, S100A4, and α-SMA [14–16], was observed in HFL-1 cells after incubating with OS-sEVs for 24 h (Fig. 4B). Additionally, in the OS-sEVs-treated cells, there was an observed upregulation of inflammatory factors like IL-8, IL-6, and IL-1β, as well as extracellular matrix (ECM) remodeling markers Collagen type I (COL1A1) and Collagen type III (COL3A1). These factors played a significant role in facilitating the formation of PMN, as shown in the right panel of Fig. 4B. However, no significant alteration was observed in these factors of HFL-1 cells following treatment with CE-sEVs for 24 h compared to control (Fig. 4B). Furthermore, previous studies have demonstrated an increased ability of activated LFs to migrate and adhere to ECM [23]. Therefore, the migration and contract ECM abilities of HFL-1 cells were assessed following their exposure to OS-sEVs or CE-sEVs. The results suggest that OS-sEVs stimulate both the migration and contraction of HFL-1 cells. Specifically, the migration rate ratio between the OS-sEVs group and the control group was measured to be 1.36 ± 0.04 at 12 h and 1.21 ± 0.02 at 24 h. The contraction rate ratio between the OS-sEVs group and the control group was determined to be 1.14 ± 0.03 at 24 h and 1.39 ± 0.06 at 72 h (Fig. 4C-F). On the other hand, the migration rate in CE-sEVs group shown no significant difference compared to the control group with a ratio of 0.92 ± 0.04 at 12 h and 0.98 ± 0.02 at 24 h. Similarly, the contraction rate in the CE-sEVs group was not significantly different from the control group, with a ratio of 0.98 ± 0.03 at 24 h and 1.09 ± 0.06 at 72 h (Fig. 4C-F).

We then examined the phenotypic variations in LFs following treatment with a higher-level concentration of CE-sEVs (1 × 10^10^ particles/mL). Surprisingly, even at such high concentrations of CE-sEVs, the LFs did not exhibit the activation tendency, as evidenced by the unchanged expression levels of activation markers (FAP, S100A4, and α-SMA), ECM remodeling markers (COL1A1 and COL3A1), and inflammatory factors (IL-8, IL-6, and IL-1β) (Fig. S3A). Additionally, the migration and contraction abilities of the LFs were comparable to those of the control group (Fig. S3B, S3C). In conclusion, the CE-sEVs exhibited a reassuring safety profile at a concentration of 1 × 10^10^ particles/ml, at which CE-sEVs wherein it failed to activate the LFs.

We further performed the RNA-seq analysis to assess the changes in gene expression of HFL-1 cells incubated with OS-sEVs or CE-sEVs. The analysis showed a significant difference gene expression profile between HFL-1 cells treated with OS-sEVs and the control group, as well as the group treated with CE-sEVs (Fig. S4A-S4D). Notably, the Kyoto Encyclopedia of Genes and Genomes (KEGG) analysis showed that compared to control group and CE-sEVs-treated group, the differentially expressed genes (DEGs) in the OS-sEVs-treated group were significantly enriched in functional annotations related to the TGF-β signaling pathway, which is closely associated with LFs activation [13, 24, 25] (Fig. S4E, S4F). This suggests that OS-sEVs may activate the LFs via the TGF-β signaling pathway, and CE-sEVs do not exhibit the same effect. These high-throughput data align with our previous in vitro findings, demonstrating that OS-sEVs can activate LFs, whereas CE-sEVs do not possess this capability.

**Fig. 5:**
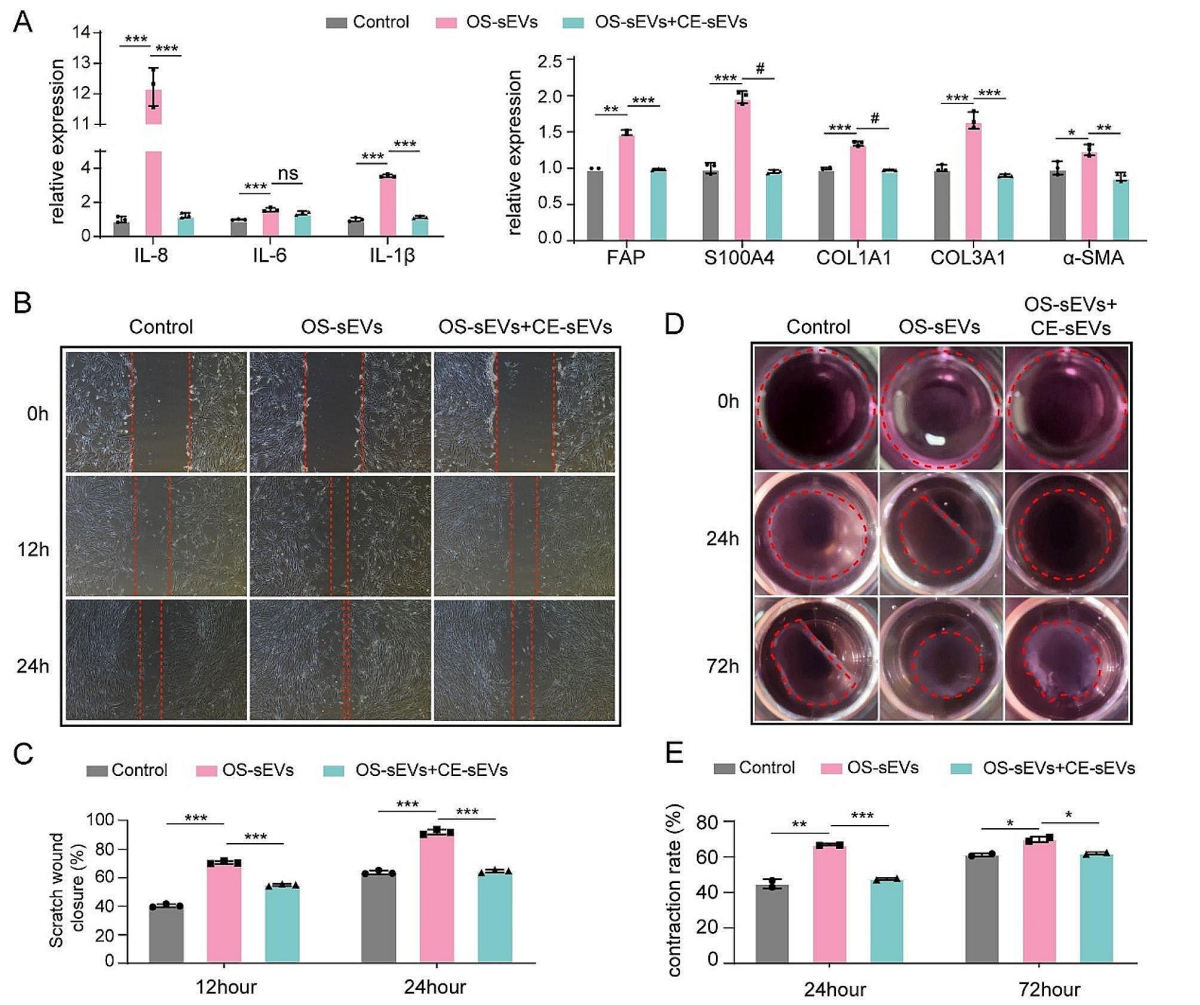
LFs activation induced by OS-sEVs efficiently suppressed by CE-sEVs. **(A)** RT-qPCR analysis the expression of the genes associated with LFs activation in HFL-1 cells treated with OS-sEVs and OS-sEVs + CE-sEVs for 24 h (*n* = 3). **(B)** Representative images of wound healing analysis of HFL-1 cells cultured with OS-sEVs or OS-sEVs + CE-sEVs for 0 h, 12 h, and 24 h. **(C)** quantification of the migration rate of HFL-1 cells cultured with OS-sEVs or OS-sEVs + CE-sEVs for 0 h, 12 h, and 24 h (*n* = 3). **(E)** Representative images of collagen matrix contraction analysis of HFL-1 cells cultured with OS-sEVs or OS-sEVs + CE-sEVs for 0 h, 24 h, and 72 h and **(F)** quantification of the contraction rate of HFL-1 cells cultured with OS-sEVs or OS-sEVs + CE-sEVs for 0 h, 24 h, and 72 h (*n* = 2). ns *P* > 0.05; * *P* < 0.05; ** *P* < 0.01; *** *P* < 0.001; # *P* < 0.0001

Due to that CE-sEVs could competitively inhibit the uptake of OS-sEVs by LFs. Subsequently, we investigated whether CE-sEVs could impede LFs activation in the presence of OS-sEVs. HFL-1 cells were treated with either OS-sEVs (1 × 10^9^ particles/mL) alone or a mixture of OS-sEVs (1 × 10^9^ particles/mL) and CE-sEVs (1 × 10^10^ particles/mL). The RT-qPCR results re-confirmed the ability of OS-sEVs in LFs activation. As shown in Fig. 5A, HFL-1 cells treated with OS-sEVs exhibit high expression levels of activated LFs markers (FAP, S100A4, and α-SMA), inflammatory markers (IL-8, IL-6, and IL-1β), and ECM remodeling markers (COL1A1, COL3A1). However, in the group treated with the mixture of OS-sEVs and CE-sEVs, these markers exhibited no significant changes, indicating the inhibition of CE-sEVs on LFs activation in the presence of OS-sEVs (Fig. 5A). Moreover, CE-sEVs also blocked the enhanced migration and contraction abilities of HFL-1 cells induced by OS-sEVs, as depicted in Fig. 5B and E. HFL-1 cells co-treated with OS-sEVs and CE-sEVs exhibited reduced migration and contraction abilities, resembling the control group, compared to the group treated solely with OS-sEVs. The migration rate ratio in the OS-sEVs + CE-sEVs group vs. OS-sEVs group was 0.78 ± 0.01, and 0.70 ± 0.01 at 12 h and 24 h, respectively. Similarly, the contraction rate ratio in the OS-sEVs + CE-sEVs group vs. the OS-sEVs group was 0.87 ± 0.01 and 0.88 ± 0.01 at 24 h and 72 h, respectively (Fig. 5B-E). These findings provide collective support for our hypothesis that CE-sEVs could inhibit the LFs activation in the presence of OS-sEVs.

### CE-sEVs induced a reduction in pulmonary metastasis by inhibiting PMN formation

**Fig. 6:**
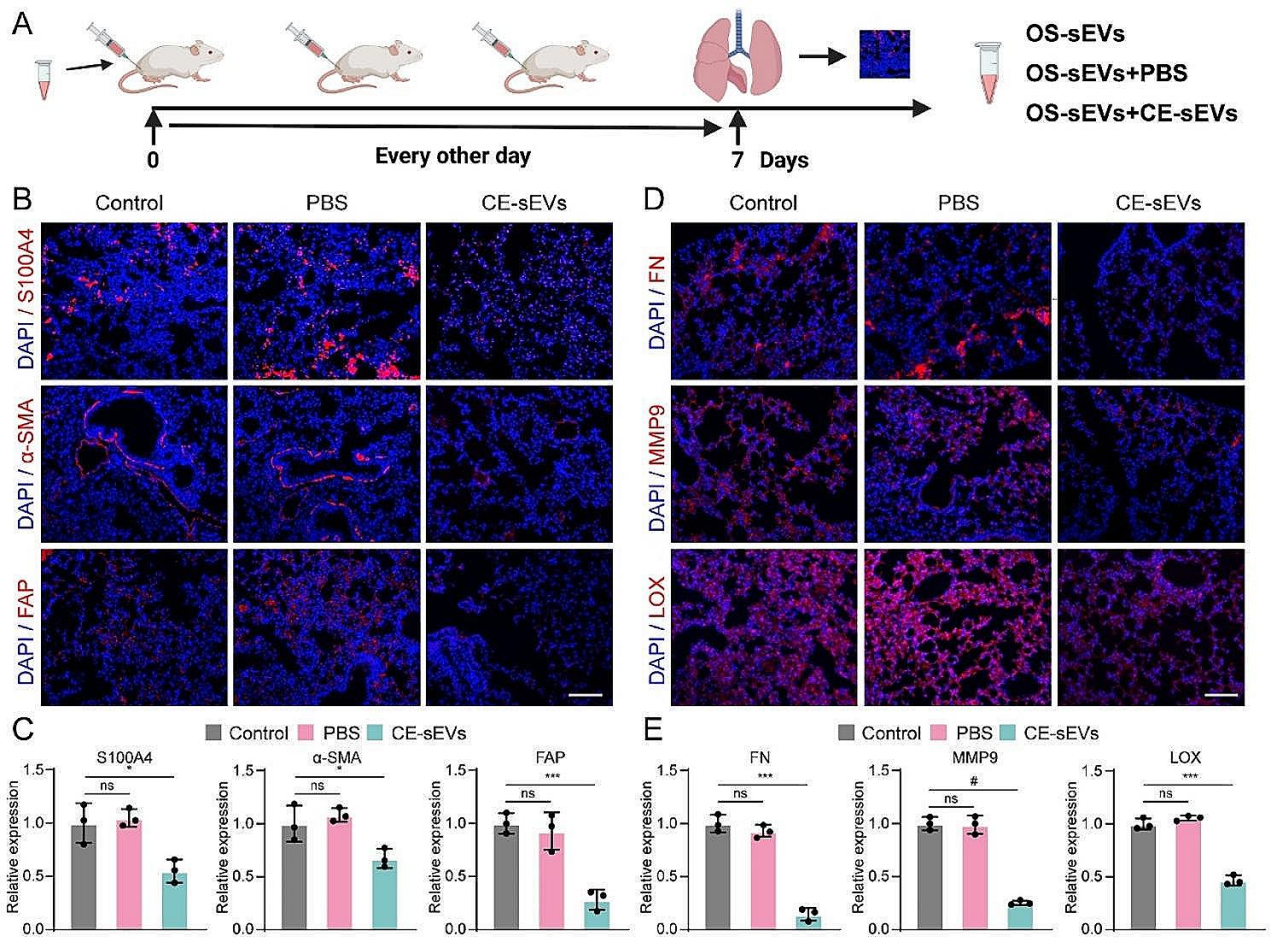
CE-sEVs mediated competitive cellular uptake suppressed LFs activation and PMN formation. **(A)** Schematic representation of the detection of activated LFs and PMN, the mice were assigned into three groups: the control group treated with 1 × 10^9^ particles OS-sEVs, the PBS group treated with 1 × 10^9^ particles OS-sEVs + PBS, and the CE-sEVs group treated with 1 × 10^9^ particles OS-sEVs + 1 × 10^10^ particles CE-sEVs. Treatments were given every other day. On day 7, lung tissues were collected follow by IF detection. **(B)** Representative IF image of LFs activation markers (S100A4, α-SMA, and FAP), scale bar: 100 μm. **(C)** Quantification of FI for LFs activation markers (S100A4, α-SMA, and FAP) (*n* = 3). **(D)** Representative IF image of PMN markers (FN, MMP9, and LOX), scale bar: 100 μm. **(E)** Quantification of FI for PMN markers (FN, MMP9, and LOX) (*n* = 3). ns *P* > 0.05; * *P* < 0.05; *** *P* < 0.001; # *P* < 0.0001

The activation of LFs is pivotal in the formation of pulmonary PMN. Thus, next, we investigated CE-sEVs’ inhibitory effects on PMN formation by attenuating LFs activation. A new set of mice was divided into three groups: the control group received an intravenous injection of 1 × 10^9^ particles OS-sEVs (100 µL), the PBS group received a mixed injection of 1 × 10^9^ particles OS-sEVs (100 µL) and PBS (100 µL), and the CE-sEVs group received a mixed injection of 1 × 10^9^ particles OS-sEVs (100 µL) and 1 × 10^10^ particles CE-sEVs (100 µL). Treatments were administered every other day (Fig. 6A). On the 7th day, lung tissues were collected to assess the activation of LFs and PMN formation (Fig. 6A). The expression levels of the well-established markers for activated fibroblasts (S100A4, α-SMA, FAP) and PMN markers such (FN, MMP9, and LOX) [26, 27] were examined by IF. The results shown in Fig. 6B-6E demonstrated that PBS group exhibited similar FI to the control group indicating that PBS do not impact the activation of LFs and the formation of PMN induced by OS-sEVs. In contrast, the CE-sEVs group displayed a significant attenuation of FI compared to the control group which received OS-sEVs treatment only (the ratio of FI in CE-sEVs group (S100A4, α-SMA, FAP, FN, MMP9, and LOX) vs. control group was 0.55 ± 0.11, 0.67 ± 0.09, 0.28 ± 0.10, 0.15 ± 0.06, 0.25 ± 0.02, and 0.47 ± 0.05, respectively). These findings suggested that the presence of CE-sEVs exerted a pronounced inhibitory effect on the activation of LFs and the formation of pulmonary PMN.

For further confirmation, we assessed these markers in an additional nude mouse model with spontaneous OS metastasis. Nude mice were injected with 1 × 10^6^ MNNG cells in the tibial medullary cavity and randomly divided into three groups (Fig. S5A). Subsequently, they were treated with either blank (control), PBS (100 µL), or CE-sEVs (1 × 10^10^ particles, 100 µL) three times a week for 14 days. On day 14, lung tissues were collected and subjected to IF analysis (Fig. S5A). Consistent results were obtained, where similar FI was observed in the control and PBS groups, indicating that OS-sEVs secreted by in situ OS cells can initiate the activation of LFs and formation of pulmonary PMN. However, the decreased FI observed in the CE-sEVs group confirmed the inhibitory effects of CE-sEVs on OS-sEVs-mediated LFs activation and pulmonary PMN development (the ratio of FI in CE-sEVs group (S100A4, α-SMA, FAP, FN, MMP9, and LOX) vs. control group was 0.68 ± 0.11, 0.37 ± 0.04, 0.14 ± 0.03, 0.07 ± 0.05, 0.13 ± 0.05, and 0.36 ± 0.08) (Fig. S5B-S5E). Collectively, these findings indicate that competitive cellular uptake mediated by CE-sEVs suppresses PMN formation in the lung.

**Fig. 7:**
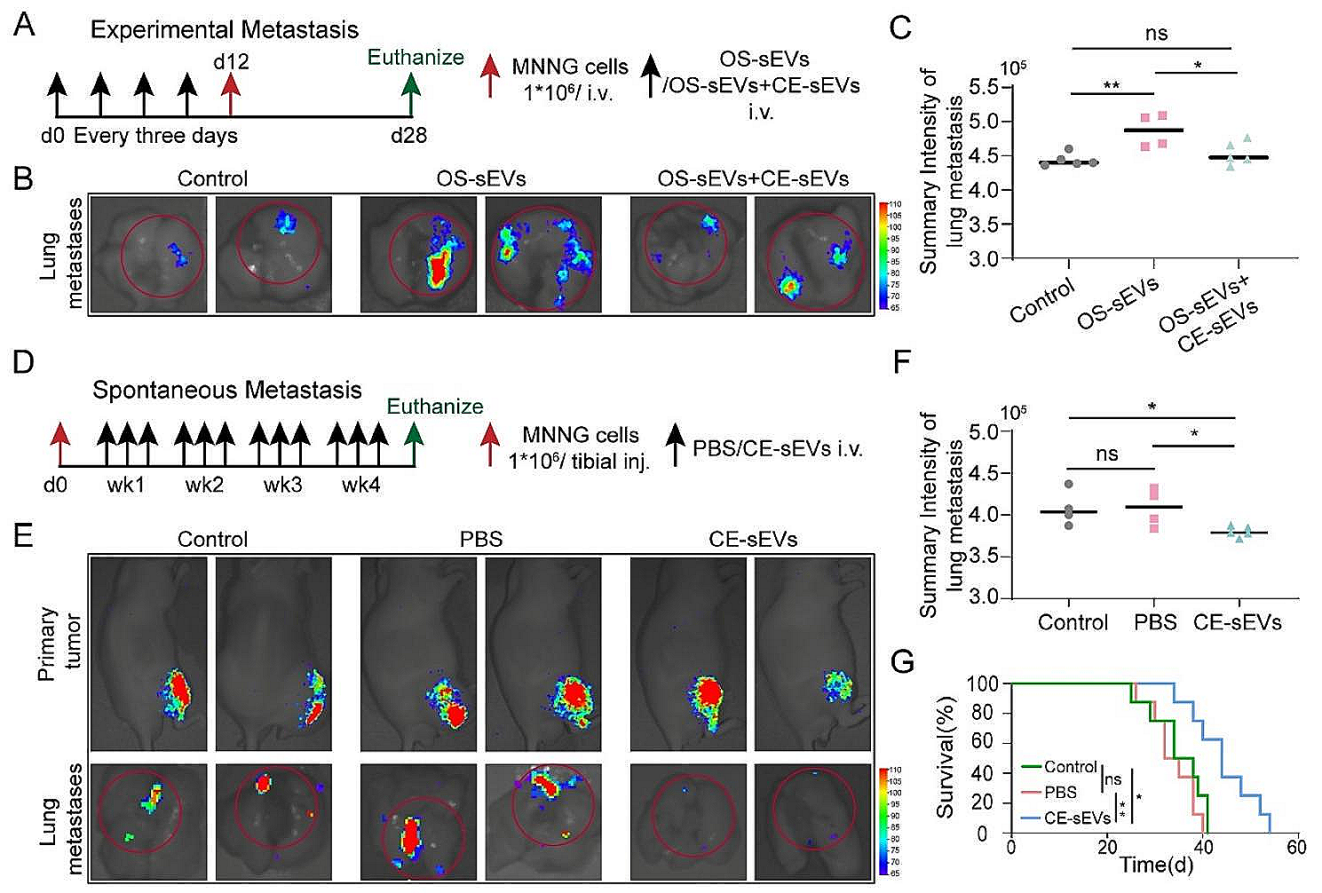
CE-sEVs efficiently suppressed the pulmonary metastasis of OS. **(A)** Schematic representation of the experimental metastasis model of OS. The mice were divided into three groups and pretreated with blank (control group), OS-sEVs (OS-sEVs group), OS-sEVs + CE-sEVs (CE-sEVs group) every three days. Then, MNNG cells were intravenous injection on day 12, mice were euthanized on day 28, and lungs were excised for observation of metastasis using BLI. **(B)** Representative ex vivo BLI of the lungs in experimental metastasis model, and the pulmonary metastasis of MNNG cells were calculated based on the lung’s FI value. **(C)** Quantification of lung’s FI in experimental metastasis model (*n* = 4/5). **(D)** Schematic representation of the spontaneous metastasis model of OS. The mice received MNNG cells inoculation into the tibia on day 0, and then divided into three groups: the control group (administered with blank), the PBS group (administered with PBS), and the CE-sEVs group (administered with CE-sEVs). Thrice-weekly interventions were performed. At the end of week 4, the mice were euthanized, and lungs were excised for observation of metastasis using BLI. **(E)** Representative ex vivo BLI of the primary tumors and lungs in spontaneous metastasis model, and the pulmonary metastasis of MNNG cells were calculated based on the lung’s FI value. **(F)** Quantification of lung’s FI in spontaneous metastasis model by BLI (*n* = 4/5). **(G)** Kaplan-Meier analysis of survival time in mice with spontaneous OS metastasis model (*n* = 8). ns *P* > 0.05; * *P* < 0.05; ** *P* < 0.01

Pulmonary PMN formation is pivotal in OS lung metastasis. Thus, we conducted investigations to assess the potential of CE-sEVs in preventing OS metastasis. In the experimental metastasis model, mice were pretreated with 1 × 10^9^ particles of OS-sEVs or a combination of 1 × 10^9^ particles OS-sEVs and 1 × 10^10^ particles CE-sEVs. Non-pretreatment mice served as the control. Each pretreatment occurred every three days, followed by intravenous injection of MNNG-luc cells on day 12. On day 28, the mice were euthanized and their lungs were excised for ex vivo observation of lung metastasis (Fig. 7A). Results revealed a significant increase in lung FI in the OS-sEVs pretreatment group (4.90 × 10^5^ ± 0.24 × 10^5^ p/s/(µW/cm^2^)) compared to the control group (4.44 × 10^5^ ± 0.10 × 10^5^ p/s/(µW/cm^2^)), indicative of increased pulmonary metastasis of OS cells with OS-sEVs pretreatment. In contrast, the FI in the co-pretreatment group decreased (4.50 × 10^5^ ± 0.17 × 10^5^ p/s/(µW/cm2)) compared to the OS-sEV pretreatment group, reaching the similar level as the control group (Fig. 7B and C, Fig. S6A). Consistent with the bioluminescence imaging (BLI) results, visual observation of hematoxylin and eosin (HE) staining indicated that pretreatment with OS-sEVs significantly enhanced lung metastasis of OS, whereas CE-sEVs counteracted the promoting effect of OS-sEVs (Fig. S6C). These results demonstrate that OS-sEVs facilitate the adhesion and growth of circulating OS cells in the lung by promoting PMN formation. However, the presence of CE-sEVs competes with OS-sEVs for cellular uptake, thereby attenuating their effects. Consequently, CE-sEVs help prevent PMN formation and pulmonary metastasis of OS cells.

The spontaneous metastasis model was established to mimic a more realistic process of OS metastasis. The mice were inoculated with MNNG-luc cells into the tibia on day 0, as depicted in Fig. 7D. Subsequently, the mice were randomly assigned to three groups for thrice-weekly interventions: the control group (administered with blank), PBS group (administered with PBS), and CE-sEVs group (administered with 1 × 10^10^ particles of CE-sEVs). The mice were euthanized at the end of week 4, and their lung were extracted to evaluate the occurrence of metastasis (Fig. 7D). Reaffirming the experimental metastasis model findings, the results indicated a significant reduction in lung FI in the CE-sEVs group (3.80 × 10^5^ ± 0.06 × 10^5^ p/s/(µW/cm^2^)) compared to the control (4.08 × 10^5^ ± 0.21 × 10^5^ p/s/(µW/cm^2^)) and PBS groups (4.08 × 10^5^ ± 0.23 × 10^5^ p/s/(µW/cm^2^)), confirmed the effective inhibition property of CE-sEVs in the development of OS pulmonary metastasis (Fig. 7E and F, Fig. S6B). The lung HE staining also showed the lower pulmonary metastasis of in CE-sEVs treatment group (Fig. S6D).

The spontaneous metastasis model was established to mimic a more realistic process of OS metastasis. The mice were inoculated with MNNG-luc cells into the tibia on day 0, as depicted in Fig. 7D. Subsequently, the mice were randomly assigned to three groups for thrice-weekly interventions: the control group (administered with blank), PBS group (administered with PBS), and CE-sEVs group (administered with 1 × 10^10^ particles of CE-sEVs). The mice were euthanized at the end of week 4, and their lung were extracted to evaluate the occurrence of metastasis (Fig. 7D). Reaffirming the experimental metastasis model findings, the results indicated a significant reduction in lung FI in the CE-sEVs group (3.80 × 10^5^ ± 0.06 × 10^5^ p/s/(µW/cm^2^)) compared to the control (4.08 × 10^5^ ± 0.21 × 10^5^ p/s/(µW/cm^2^)) and PBS groups (4.08 × 10^5^ ± 0.23 × 10^5^ p/s/(µW/cm^2^)), confirmed the effective inhibition property of CE-sEVs in the development of OS pulmonary metastasis (Fig. 7E and F, Fig. S6B). The lung HE staining also showed the lower pulmonary metastasis of in CE-sEVs treatment group (Fig. S6D).

Moreover, the survival rate of mice with spontaneous metastasis was statistically analyzed using the Kaplan-Meier method. The OS-bearing mice in the CE-sEVs group exhibited significantly prolonged survival (average 44.25 ± 6.88 days) compared to the PBS group (average 33.88 ± 4.73 days) and the control group (average 35.88 ± 5.79 days) (Fig. 7G). Finally, HE staining of major organs was performed to evaluate the potential toxicity of OS-sEVs and CE-sEVs in vivo. As depicted in Fig. S7A, S7B, no significant toxicity towards major organs was observed in either OS-sEVs or CE-sEVs groups.

## Discussion

OS is one of the most aggressive cancers diagnosed in teenagers. Up to now, the 5-year survival rate of the localized OS can be over 60% due to the sequential comprehensive therapy: preoperative chemotherapy, tumor removal, and postoperative chemotherapy [2]. However, pulmonary metastasis reduces the survival rate of OS patients to less than one-third of those without metastasis, becoming the leading cause of OS-related death [1]. Remodeling of the local lung microenvironment into PMN which supports the colonization and proliferation of tumor cells is an indispensable condition for the pulmonary metastasis of OS [4, 5, 28]. In the present study, we found that CE-sEVs show reduced pro-tumoral and LFs activating ability but retain similar physicochemical properties and innate targeting of LFs as OS-sEVs. Then, we further confirm that CE-sEVs could suppress the PMN formation in lung and the pulmonary metastasis of OS. These results demonstrated for the first time that CE-sEVs possess the anti-metastasis ability in OS by regulating PMN formation.

PMN was initially proposed by Kaplan et al. in 2005 as microenvironments within metastatic organs or tissues that facilitate tumor cell infiltration and subsequent secondary metastasis [4, 28]. Therefore, there has been a surge of interest in inhibiting such metastasis by blocking PMN formation [29, 30]. For example, targeting lung PMN with siRNA-loaded sEVs, and then regulating PMN formation and inhibiting breast cancer pulmonary metastasis [31]. Utilizing myeloid cells to deliver IL-12 to lung, activating antigen presentation cells and T cells, thereby altering the local immunosuppressive environment of PMN and inhibiting the pulmonary metastasis of rhabdomyosarcoma [32]. Co-delivering chemotherapy drugs and immunomodulatory agents by neutrophil membrane to alleviate the immunosuppressive state of lung PMN, reduce vascular permeability, and inhibit the pulmonary metastasis of breast cancer [33]. While these methods have produced unexpected results, the complexity of constructing and modifying intricate delivery platforms to improve PMN targeting and inhibition has always been a challenge. Therefore, we aim to develop a more straightforward approach to inhibit PMN formation in the lung.

The process of PMN formation involves multiple steps, with particular emphasis on the involvement of primary tumor-derived components and the remodeling of extracellular matrix microenvironment at metastatic sites [34, 35]. The activation of fibroblasts into CAFs served as one of the most significant events during the PMN formation [34]. Recent studies show that tumor-derived sEVs (TsEVs), an important communication medium between primary tumors and metastatic sites, could reprogram the fibroblasts in the lung and promote PMN construction [13, 36]. Hoshino et al. found that distinct integrin expression confers organ-specific targeting to TsEVs, and co-localization of lung-targeting sEVs with LFs was observed within the pulmonary milieu [12]. Given the crucial role of sEVs uptake by fibroblasts in the process of PMN formation and tumor metastasis, blocking it may effectively impede tumor metastasis [37–40]. Our previous research has revealed a straightforward approach for modifying glioma-derived sEVs into cargos-eliminated sEVs using saponin, the cargos-eliminated sEVs retaining their inherent targeting ability while lacking pro-tumoral potential [20]. Based on these, we introduced the concept of “competitive inhibition” to hinder the the activation of LFs by OS-sEVs, that is, developing CE-sEVs that target LFs but do not activate LFs, and using them as competitors to inhibit the internalization of OS-sEVs by LFs, thereby preventing the activation of LFs and the formation of PMN.

In the present study, we eliminated most of the contents from OS-sEVs through saponin treatment, with an elimination efficiency of approximately 50-60% for proteins and over 75% for RNAs. This can be attributed to the fact that while proteins are distributed not only on but also within the membrane, RNAs predominantly exist within the membrane, thereby resulting in its superior removal efficacy. The CE-sEVs were further confirmed to show no obvious differences from OS-sEVs in the physicochemical properties, including appearance, size, and the expression of characteristic markers. Similar to our previous research findings, we observed that CE-sEVs exhibit reduced pro-tumoral and LFs activation ability, while retaining a similar innate targeting of LFs as OS-sEVs. These data demonstrated the biosafety of CE-sEVs in their application and suggest that they may serve as a competitive substrate for LFs uptake of OS-sEVs.

Considering that the absorption of sEVs by cells is not unlimited but rather follows a saturation pattern, we are further concerned about the inhibitory capacity of CE-sEVs on the uptake of OS-sEVs by LFs. An inverse correlation between the number of OS-sEVs uptake by LFs and the concentration of CE-sEVs was observed. According to our results, high concentrations of CE-sEVs (10 times the OS-sEVs) can reduce the uptake of OS-sEVs by LFs in vitro by approximately 80% and decrease the accumulation of OS-sEVs in the lung by about 50%. More importantly, due to the significant suppression of uptake of OS-sEVs by LFs, CE-sEVs can attenuate the activation of LFs and the formation of PMN in lung, ultimately suppressing the lung metastasis of OS cells. In summary, our findings suggest a novel strategy for preventing OS lung metastasis by inhibiting PMN formation through the use of CE-sEVs, obviating the need for additional complex modifications. To the best of our knowledge, there is limited research assessing the preventive function of unmodified cargos-eliminated sEVs in tumor metastasis. We provide first-hand evidence that CE-sEVs might be of great value in preventing OS pulmonary metastasis and this warrants further clinical trials.

## Conclusion

**Fig. 8:**
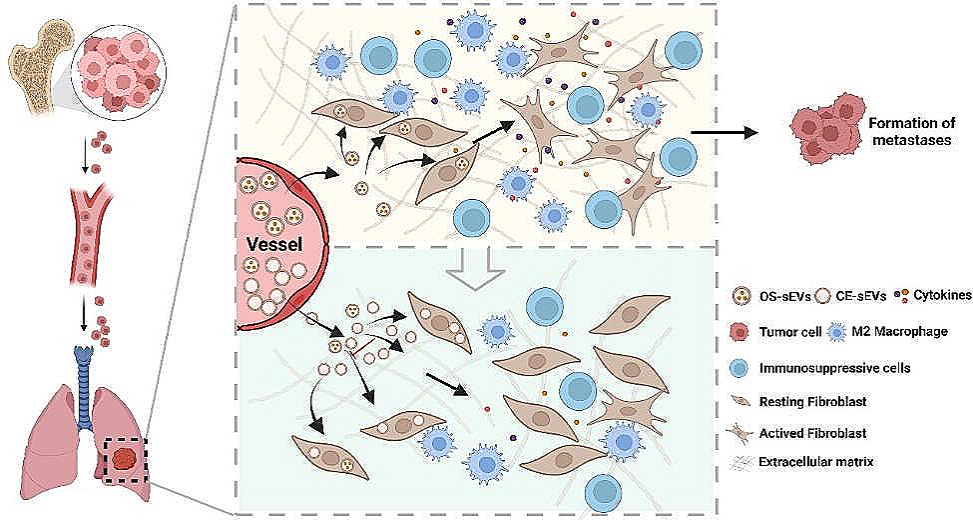
Schematic illustration of the mechanisms how CE-sEVs prevent the formation of PMN formation in lung induced by OS-sEVs. CE-sEVs inhibit the LFs activation by competitively blocking the uptake of OS-sEVs. This inhibition ultimately prevented the modification of the local pulmonary microenvironment, resulting in the suppression of PMN formation and pulmonary metastasis of OS.

In summary, by eliminating original cargos of OS-sEVs through saponin treatment, a non-tumor promoting and non-LFs activating subtype of OS-sEVs (CE-sEVs) is obtained. CE-sEVs possess similar targeting abilities for LFs as OS-sEVs, and can effectively competing and reducing the uptake of OS-sEVs by LFs. In vitro and in vivo experiments confirmed that the massive co-existed of CE-sEVs inhibits OS-sEVs-induced LFs activation and lung PMN formation through competitive cellular uptake strategies. Furthermore, in both experimental and spontaneous metastasis OS models in mice, CE-sEVs treatment reduced the lung metastasis of OS cells (Fig. 8). This study provides an intervention strategy for preventing LFs activation, pulmonary PMN formation, and OS lung metastasis through the competitive inhibition of OS-sEVs function by CE-sEVs.

### Electronic supplementary material

Below is the link to the electronic supplementary material.

Supplementary Material 1

Supplementary Material 2

## Data Availability

No datasets were generated or analysed during the current study.
