# Supplementary material for: Cargo-eliminated osteosarcoma-derived small extracellular vesicles mediating competitive cellular uptake for inhibiting pulmonary metastasis of osteosarcoma
Source: J Nanobiotechnology. 2024 Jun 22;22:360. doi: 10.1186/s12951-024-02636-9 (PMC11193292; doi:10.1186/s12951-024-02636-9)
Supplement: Supplementary file 1 — Supplementary Material 1 [file 12951_2024_2636_MOESM1_ESM.docx]

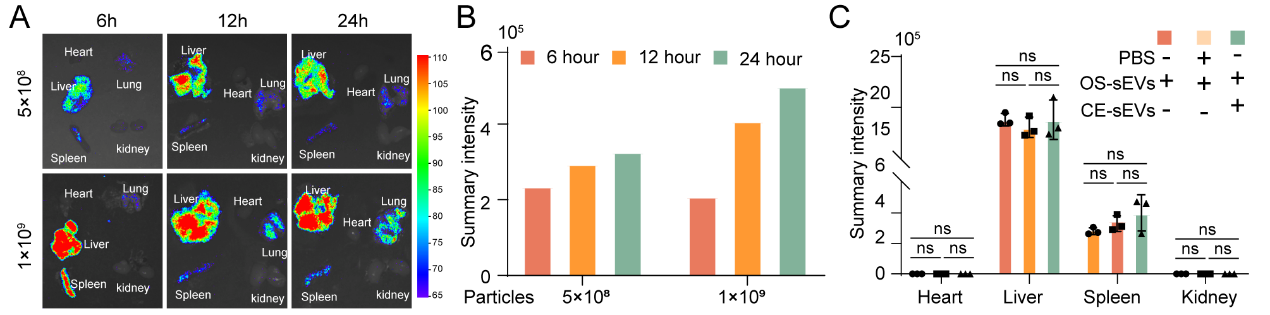


**Fig. S1 (A)** Representative *ex vivo* BLI of OS-sEVs uptake at different concentrations by major organs at different time points, and **(B)** bar graph illustrates the total lung FI. **(C)** Statistical analysis of the FI in heart, liver, spleen, and kidney (n=3).


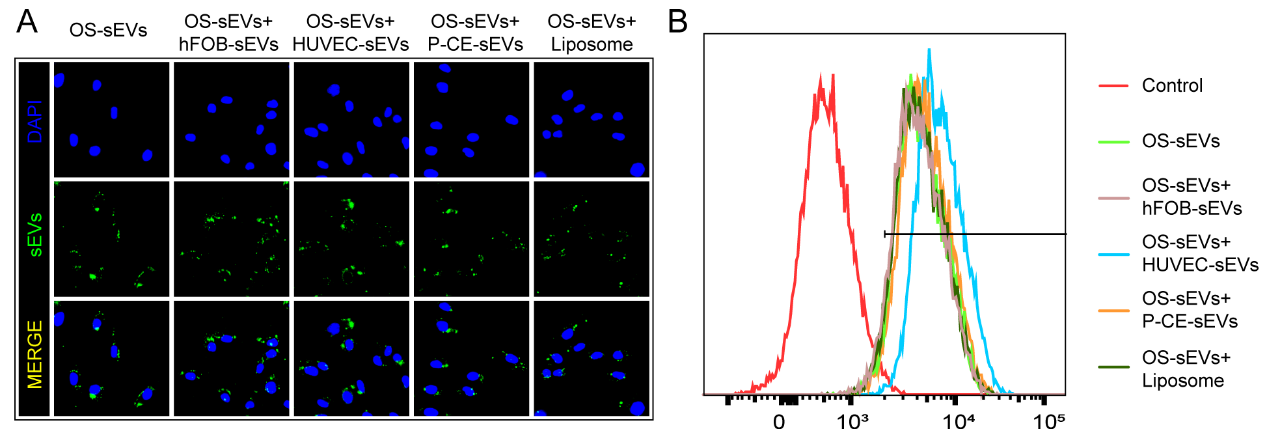


**Fig. S2** **(A)** IF show the uptake efficiency of HFL-1 towards OS-sEVs in different treatment condition (non-treatment, 1×10^10^ particles/mL of hFOB-sEVs, 1×10^10^ particles/mL HUVEC-sEVs, 1×10^10^ particles/mL P-OS-sEVs and 1×10^10^ particles/mL liposome). **(B)** Flow cytometry show the uptake efficiency of HFL-1 towards OS-sEVs in different treatment condition (non-treatment, 1×10^10^ particles/mL of hFOB-sEVs, 1×10^10^ particles/mL HUVEC-sEVs, 1×10^10^ particles/mL P-OS-sEVs and 1×10^10^ particles/mL liposome).


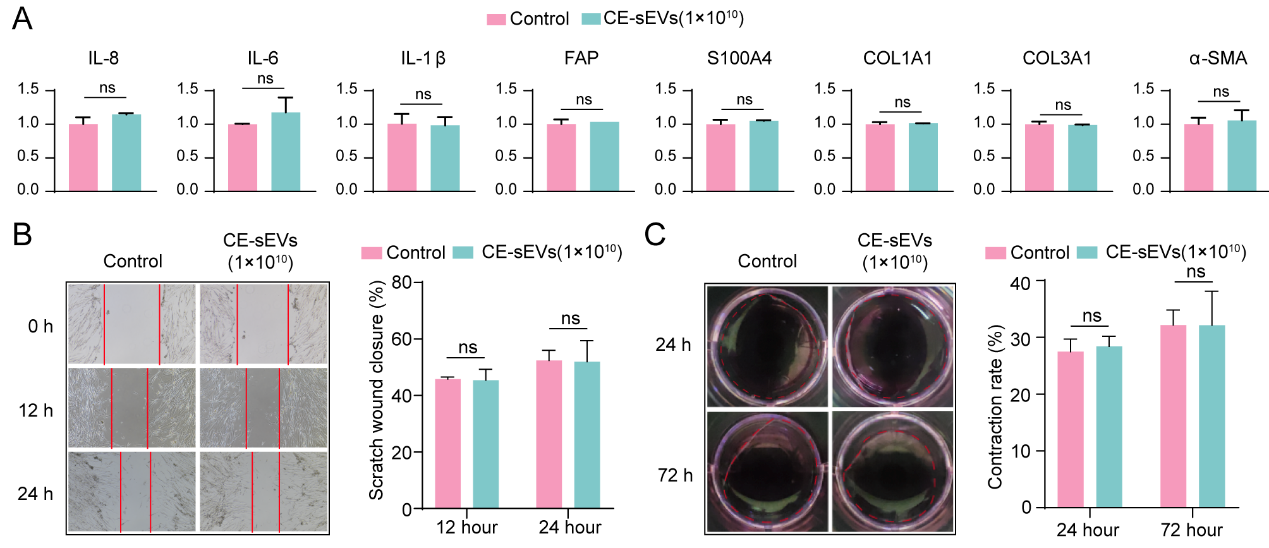


**Fig. S3 (A)** RT-qPCR analysis the expression of the genes associated with LFs activation in HFL-1 cells treated with blank or 1×10^10^ particles/ml of CE-sEVs for 24 h. **(B)** Representative images of wound healing analysis of HFL-1 cells cultured with blank or 1×10^10^ particles/ml of CE-sEVs for 0 h, 12 h, and 24 h, and the quantification of the migration rate of HFL-1 cells cultured with blank or 1×10^10^ particles/ml of CE-sEVs. **(C)** Representative images of collagen contraction analysis of HFL-1 cells cultured with blank or 1×10^10^ particles/ml of CE-sEVs for 24 h and 72 h and the quantification of the contraction rate of HFL-1 cells cultured with blank or 1×10^10^ particles/ml of CE-sEVs. ns P>0.05.


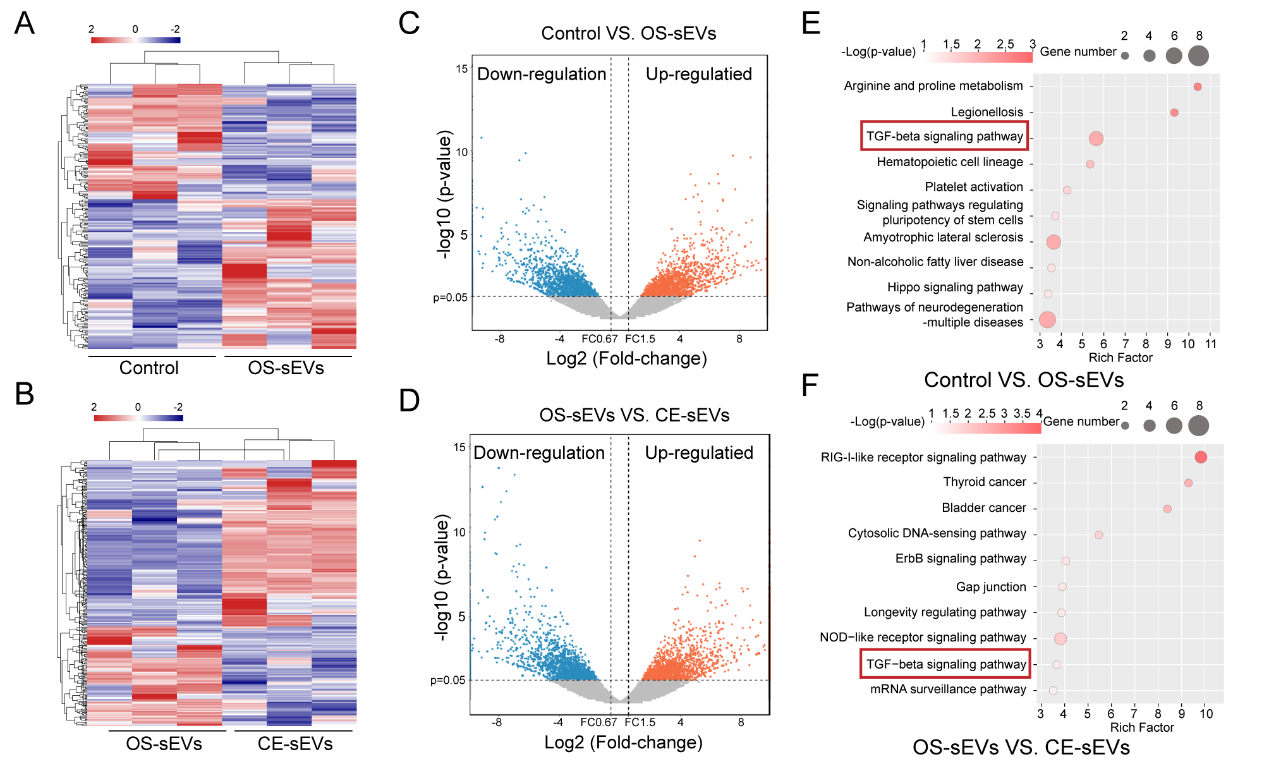


**Fig. S4** **(A, B)** Heat map represents significantly DEGs between control group and OS-sEVs group (A), OS-sEVs group and CE-sEVs group (B). **(C, D)** Volcano plots represent the DEGs between control group and OS-sEVs group (C), OS-sEVs group and CE-sEVs group (D), Fold-change values on the abscissa were log2-transformed, and P values on the ordinate were -log10 transformed. The selection criteria of DEGs were P-value < 0.05 and changed expression higher than 1.5- or lower than 0.67-fold. **(E, F)** KEGG analysis for DEGs between control group and OS-sEVs group (E), OS-sEVs group and CE-sEVs group (F), the DEGs in the OS-sEVs group were significantly enriched in functional annotations related to the TGF-β signaling pathway, which is closely associated with LFs activation.


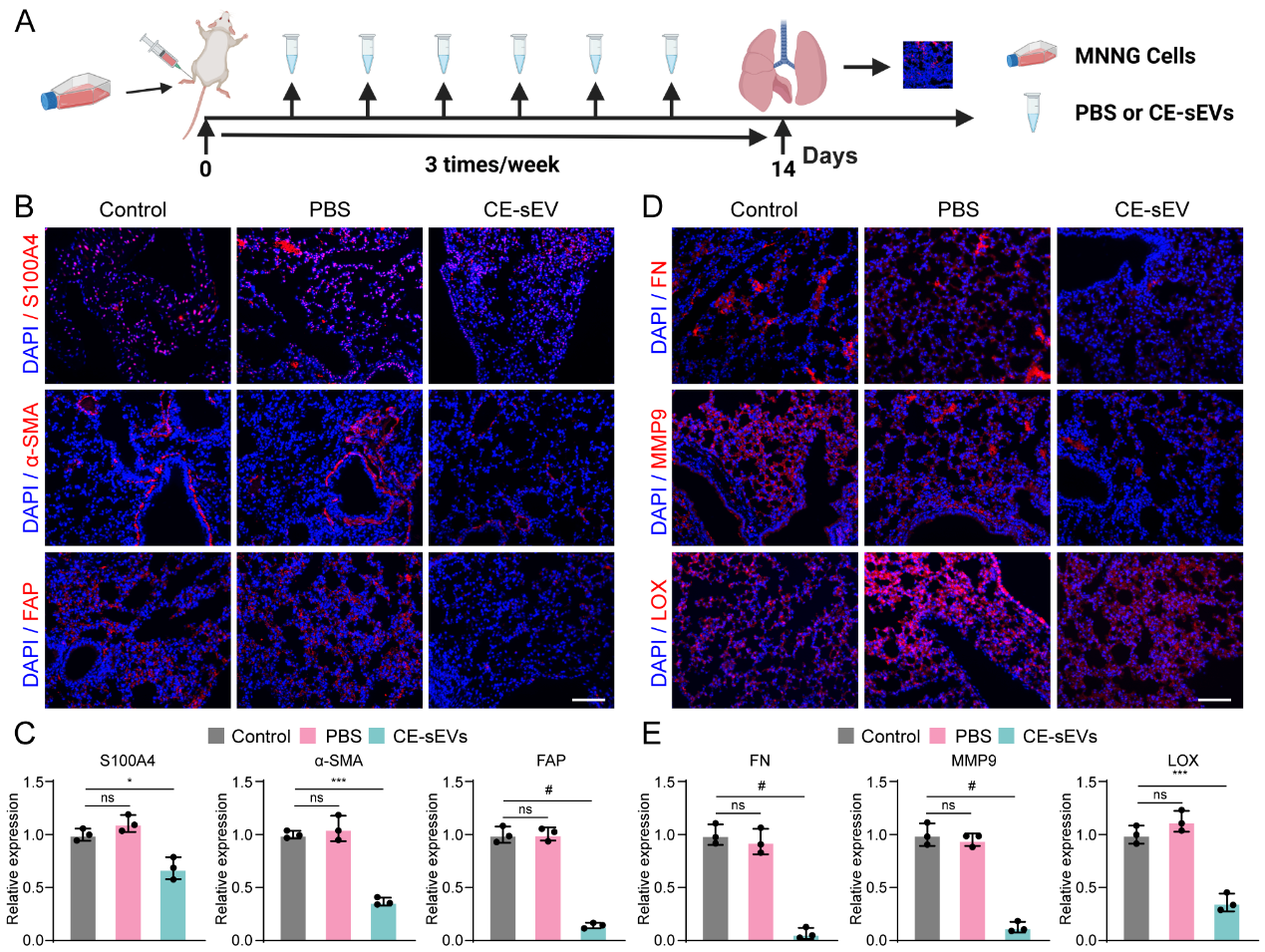


**Fig. S5 (A)** Schematic representation of the detection of activated LFs and PMN, the mice were injected with 1×10^6^ MNNG cells in the tibial medullary cavity and treated with either blank, PBS, or 1×10^10^ particles CE-sEVs three times a week for 14 days. On day 14, lung tissues were collected and subjected to IF analysis **(B)** Representative IF image of LFs activation markers (S100A4, α-SMA, and FAP), scale bar: 100 μm. **(C)** Quantification of FI for LFs activation markers (S100A4, α-SMA, and FAP) (n=3). **(D)** Representative IF image of PMN markers (FN, MMP9, and LOX), scale bar: 100 μm. **(E)** Quantification of FI for PMN markers (FN, MMP9, and LOX) (n=3). ns P>0.05; * P<0.05; *** P<0.001; # P<0.0001.


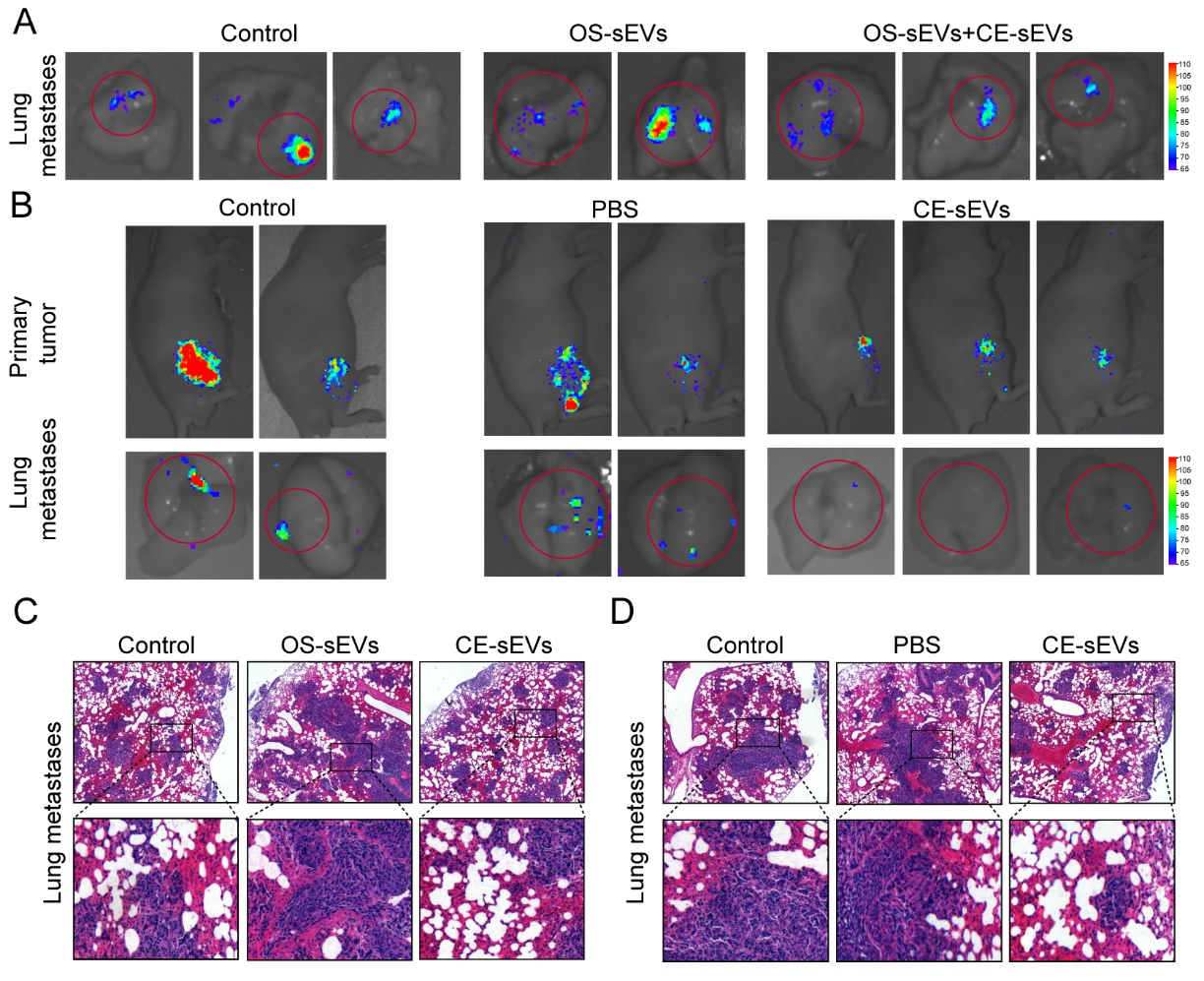


**Fig. S6 (A)** Representative *ex vivo* BLI of the lungs in experimental metastasis model, and the pulmonary metastasis of MNNG cells were calculated based on the lung’s FI value. **(B)** Representative *ex vivo* BLI of the primary tumors and lungs in spontaneous metastasis model, and the pulmonary metastasis of MNNG cells were calculated based on the lung’s FI value. **(C)** Representative HE stained images of the lungs in experimental metastasis model. **(D)** Representative HE stained images of the lungs in spontaneous metastasis model. **(E)** Representative HE stained images of the main organs including heart, liver, and kidney.


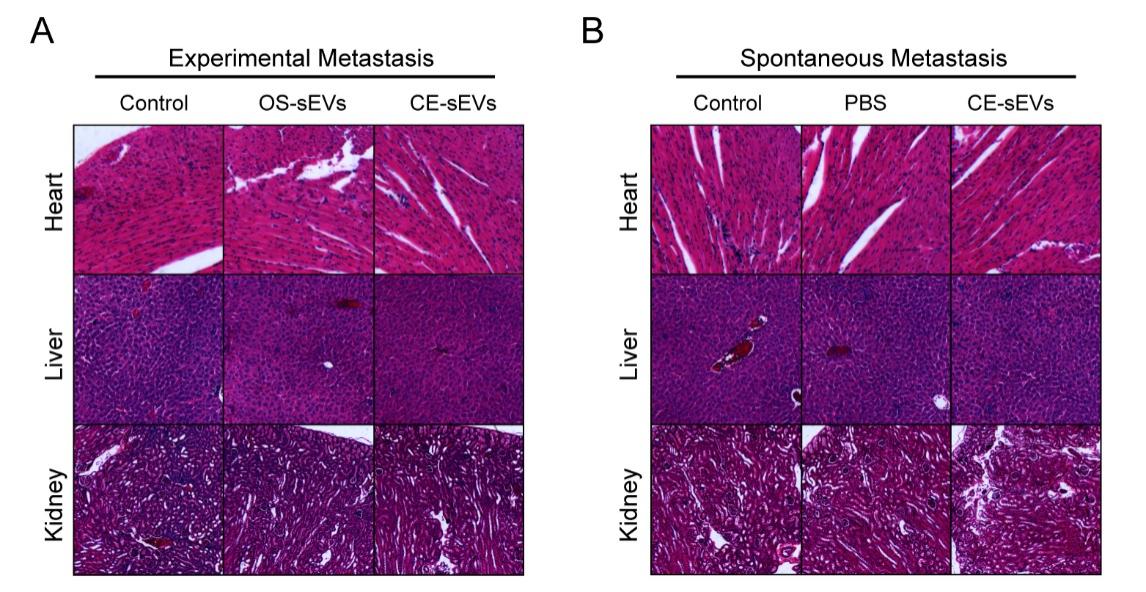


**Fig. S7 (A)** Representative HE stained images of the main organs including heart, liver, and kidney in experiment metastasis model. **(B)** Representative HE stained images of the main organs including heart, liver, and kidney in spontaneous metastasis model.
